# The Protein Phosphatase Inhibitor LB100 Targets the Mesenchymal Lineage of Pancreatic Ductal Adenocarcinoma

**DOI:** 10.1002/mco2.70794

**Published:** 2026-06-07

**Authors:** Janine Murr, Carolin Schneider, Ningjun Duan, Hazal Köse, Anantharamanan Rajamani, Xueyang He, Jonas Buchloh, Christian Hintze, Atharva Naik, Daniel Goeke, Nicole Rjasanow, Lukas Krauß, Alexandra Nguyen, Sebastian A. Widholz, Christian Schneeweis, Riccardo Trozzo, Felix Orben, Sebastian Mueller, Rupert Öllinger, Juan J Montero, Michael Dudek, Percy Knolle, Bo Kong, Volker Ellenrieder, Constanza Tapia Contreras, Elisabeth Hessmann, Marian Grade, Michael Ghadimi, Christian J. Braun, Roland Rad, Maximillian Reichert, Ulrich Keller, Roland M. Schmid, Paul L. Boutz, Dieter Saur, Matthias Wirth, Oliver H. Krämer, Günter Schneider

**Affiliations:** ^1^ Medical Clinic and Polyclinic II Klinikum Rechts Der Isar Technical University Munich Munich Germany; ^2^ Department of General Visceral and Pediatric Surgery University Medical Center Göttingen Göttingen Germany; ^3^ Department of Hematology Oncology and Cancer Immunology Campus Benjamin Franklin Charité‐Universitätsmedizin Berlin Corporate Member of Freie Universität Berlin and Humboldt‐Universität zu Berlin Berlin Germany; ^4^ Institute For Translational Cancer Research and Experimental Cancer Therapy Technical University Munich Munich Germany; ^5^ Department of Biochemistry and Biophysics University of Rochester School of Medicine and Dentistry Rochester New York USA; ^6^ Center For RNA Biology Rochester New York USA; ^7^ Wilmot Cancer Institute Rochester New York USA; ^8^ Institute of Toxicology University of Mainz Medical Center Mainz Germany; ^9^ Institute of Molecular Oncology and Functional Genomics TUM School of Medicine Technische Universität München Munich Germany; ^10^ Institute of Molecular Immunology and Experimental Oncology University Hospital München rechts der Isar Technical University of Munich München Germany; ^11^ Department of General Visceral and Transplantation Surgery Heidelberg University Hospital Heidelberg Germany; ^12^ University Medical Center Göttingen Department of Gastroenterology, Gastrointestinal Oncology and Endocrinology Göttingen Germany; ^13^ Clinical Research Unit 5002 KFO5002 University Medical Center Göttingen Göttingen Germany; ^14^ CCC‐N (Comprehensive Cancer Center Lower Saxony) Göttingen Germany; ^15^ Department of Pediatrics Dr. Von Hauner Children's Hospital University Hospital LMU Munich Munich Germany; ^16^ German Cancer Research Center (DKFZ) and German Cancer Consortium (DKTK) Heidelberg Germany; ^17^ DEFEAT‐PDAC – Decoding and Targeting the PDAC Ecosystem – A German Pancreatic Cancer Alliance (GPCA) Consortium – Partner Site Munich; ^18^ Translational Pancreatic Research Cancer Center Medical Clinic and Polyclinic II Klinikum Rechts Der Isar Technical University Munich Munich Germany; ^19^ Center For Organoid Systems (COS) TUM Garching Germany; ^20^ Max Delbrück Center (MDC) Berlin Germany; ^21^ DEFEAT‐PDAC – Decoding and Targeting the PDAC Ecosystem – A German Pancreatic Cancer Alliance (GPCA) Consortium – Partner Site Göttingen

**Keywords:** cyclin‐dependent kinase 9, mesenchymal, pancreatic cancer, protein phosphatase 2A, transcription

## Abstract

Pancreatic ductal adenocarcinoma (PDAC) remains a therapeutic challenge, and the aggressive basal‐like/mesenchymal subtype is particularly refractory to chemotherapy, underscoring the need for novel therapies. Leveraging genetic screens, we identified protein phosphatase 2A (PP2A) catalytic subunit PPP2CA as a target. Pharmacological PP2A inhibition selectively impaired the growth of mesenchymal PDAC cells. To delineate the mechanisms underlying sensitivity to the PP2A inhibitor LB100, we employed a dual‐pronged strategy. Functional characterization revealed metabolic reprogramming coupled with endoplasmic reticulum (ER) stress and cell death induction. Genome‐wide genetic screens identified key modifiers of LB100 sensitivity, implicating transcriptional regulators, mRNA processing, translation, and metabolism. Based on expression data linking PP2A to splicing and transcriptional regulation, we prioritized these processes for validation. Mesenchymal PDAC cells exhibited enhanced splicing following PP2A inhibition. Notably, we identified enhanced transcriptional elongation upon LB100 treatment, particularly of short genes, driven by cyclin‐dependent kinase 9 (CDK9). Our findings support a reciprocal regulatory relationship between PP2A and CDK9 that connects to the activation of ER stress response factors, including activating transcription factor 4 (ATF4). These results establish PP2A as a druggable target in mesenchymal PDAC cells and reveal a role of LB100‐induced transcriptional elongation and splicing, providing a mechanistic basis to guide future therapy development.

## Graphical abstract

To define the mechanisms of sensitivity to PP2A inhibitors (PP2Ai), we combined molecular profiling of PP2Ai responses with genome‐wide genetic screens. PP2Ai preferentially target mesenchymal pancreatic ductal adenocarcinoma (PDAC) cells and engage pathways linked to transcriptional elongation, splicing, metabolism, and ER stress response factors.

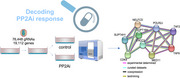

## 1 Introduction

The incidence and mortality rates of pancreatic ductal adenocarcinoma (PDAC) are rising, with a trend for an increasing incidence in individuals under the age of 50 years [1]. At the same time, the prognosis for patients with PDAC remains poor with a 5‐year survival of 13% [2]. The current standards of care for locally advanced or metastatic PDACs are modestly effective chemotherapy combinations [3]. PDAC is broadly classified into two principal molecular subtypes, classical and basal‐like, with the latter overlapping with a described mesenchymal subtype [4, 5, 6]. The basal‐like/mesenchymal subtype of PDAC demonstrates increased aggressiveness, a higher propensity for metastasis, and is associated with reduced benefit from chemotherapy, such as the FOLFIRINOX regimen [7, 8, 9, 10]. Thus, developing improved and precise therapies, gaining a deeper understanding of the molecular parameters that determine treatment responses, and prioritization of therapeutically appropriate targets are urgent medical needs.

A way to achieve these goals relies on a deeper understanding of protein phosphorylation. Phosphorylation plays a role in all hallmarks of cancers and accordingly, inhibitors of kinases revolutionized cancer therapy. Exploring the potential of phosphatases as druggable cancer targets is still an emerging field [11]. Current clinical trials test inhibitors of the tyrosine‐phosphatase Src homology region 2 domain‐containing phosphatase‐2 and PP2A [11].

PP2A, a major contributor of serine/threonine phosphatase activity in cells, is a trimeric complex composed of the scaffolding A subunit (PP2A‐A), a catalytically active C subunit (PP2A‐C), and various regulatory B subunits (PP2A‐B) [12, 13]. Depending on the context, PP2A conducts tumor‐suppressive as well as tumor‐promoting functions [11]. Well‐characterized targets of canonical PP2A complexes include oncoproteins, like the AKT kinase or the transcription factor MYC [13]. Inhibition of PP2A in cancer cells by endogenous proteins, including cancerous inhibitor of protein phosphatase 2A and the SET nuclear oncogene (I2PP2A, inhibitor 2 of PP2A, or TAF‐I), contributes to the activation of these oncoproteins [14]. As a result, PP2A‐activating drugs, including OP449 and FTY720, were established [13].

Nonetheless, accumulating evidence indicates that the documented tumor‐promoting functions of PP2A [13, 15] are pharmacologically exploitable targets in cancer cells. Therefore, PP2A‐inhibiting drugs such as LB100 have been developed and are currently in clinical trials [11]. The target spectrum of the cantharidin derivative LB100 includes PP2A and PP5 [16, 17]. LB100 has been suggested to act as a prodrug that hydrolyzes to generate the active metabolite endothall, a phosphatase inhibitor reported to selectively inhibit PP2A over PP1, PP5, or DUSP22 phosphatases [18]. In a Phase I clinical trial, LB100 demonstrated tolerability, and the only partial response was documented in a PDAC patient [19]. LB100 is currently clinically tested in myelodysplastic syndromes (NCT03886662) and in combination with immune checkpoint blockade in metastatic colorectal cancer (LB100 + atezolizumab; NCT06012734) and ovarian clear cell carcinoma (LB100 + dostarlimab; NCT06065462).

The path from target discovery to clinical implementation of cancer therapies is characterized by substantial failure rates. Systematic genome‐wide genetic screening allows unbiased functionalization of cancer genomes. Integration of gene loss‐of‐fitness effects with genomic, transcriptomic, and tractability data can be used to compute prioritization scores in a context‐dependent manner [20]. These scores allow focusing on relevant targets. In this study, we demonstrate that PP2A is a relevant target in PDAC cell lineages with mesenchymal features and we decode the molecular mechanisms that lead to cell death of PP2A inhibitor (PP2Ai)‐sensitive PDAC cells.

## 2 Results

### 2.1 PP2A is a Therapeutic Target in PDAC

**FIGURE 1:**
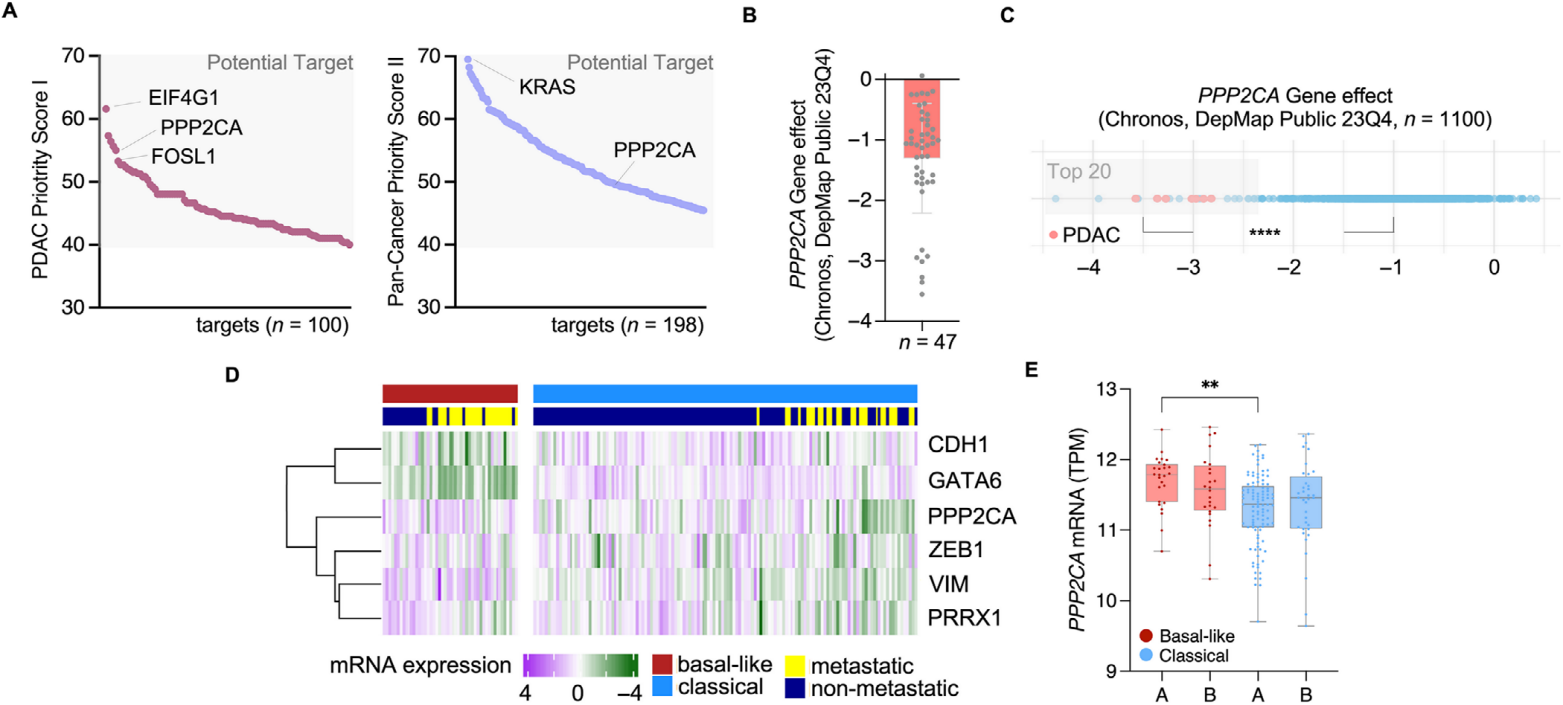
PP2A is a target in mesenchymal PDAC cells. (A) Depicted are the priority scores from the Project Score Portal with a priority score threshold >40 (gray box). *Left*: Project Score I, PDAC specific targets; *Right*: Project Score II: pan cancer targets. (B) *PPP2CA* gene effects of a CRISPR–Cas9 drop‐out score (Chronos) for *n* = 47 PDAC cell lines were retrieved via the DepMap portal (DepMap Public 23Q4). (C) Depiction of the *PPP2CA* gene effect of all cell lines (*n* = 1100) of the DepMap portal. Seven of the Top20 are from PDAC origin (red) (DepMap Public 23Q4). Please note that only PDAC lines belonging to the Top20 are labeled in red. Chi‐square: ****p Value < 0.0001. (D) Expression dataset from annotated primary and metastatic tumors were accessed from Chan‐Seng‐Yue et al. [7]  (Ontario Institute for Cancer Research). Variance‐scaled heatmap of the mRNA expression (clustering method = ward.D, clustering distance rows = euclidean) from the indicated genes are depicted. (E) Comparison of the *PPP2CA* mRNA expression from (D) in the different subtypes of PDAC (**p = 0.0024, one‐way ANOVA, Basal‐A: *n* = 26, Basal‐B: *n* = 27, Classical‐A: *n* = 51, Classical‐B: *n* = 36).

We accessed the project Score I database to define therapeutic vulnerabilities of PDAC cells [20]. Priority scores integrate gene effects from CRISPR–Cas9 genetic screens, genomic biomarkers, and target tractability. Interestingly the catalytic subunit of the PP2A phosphatase, *PPP2CA*, is among the top‐ranked PDAC‐specific priority targets (Figure 1A). We also detected additional targets, including the translational regulator EIF4G1, targets with proven activity in preclinical PDAC models, like cyclin‐dependent kinase (CDK)4 [21], or relevant transcription factors, like FOSL1 [22, 23] (Figure 1A and Table S1). The Score II dataset, which incorporates protein–protein interaction networks into target score calculations [24], coherently identifies PP2A as a pan‐cancer target (Figure 1A).

To scrutinize the significance of PP2A as a target in PDAC, we analyzed the DepMap database [25]. Here, a distinct population of PDAC cell lines experiences a significant loss of fitness following the *PPP2CA* knock‐out (Figure 1B). Seven of the 20 cell lines with the lowest *PPP2CA* loss‐of‐fitness scores in the 1100‐line Chronos 23Q4 dataset are PDAC lines (Figure 1C). Collectively, these findings highlight PP2A as a therapeutically relevant target in PDAC.

The distinctive molecular profile of basal‐like/mesenchymal PDACs is characterized by the expression of mesenchymal marker genes such as *VIMENTIN* (*VIM*), accompanied by the reduced expression of epithelial genes, including *CDH1* or *GATA6* (Figure 1D). A more detailed PDAC classification further subdivides the major subtypes into classical A/B and basal‐like A/B groups [7]. Whereas classical A/B tumors are more commonly associated with earlier disease stages, basal‐like A tumors are enriched in Stage IV metastatic PDAC [7]. Although classical PDAC cases with high *PPP2CA* mRNA expression exist (Figure 1D), its expression is significantly higher in basal‐like A tumors (Figure 1E) [7]. This is further reinforced by the reported association of high *PPP2CA* mRNA levels with worse survival [26], in summary disclosing an association between PPP2CA levels and the aggressiveness of PDAC.

### 2.2 Mesenchymal PDACs Are Sensitive to LB100

**FIGURE 2:**
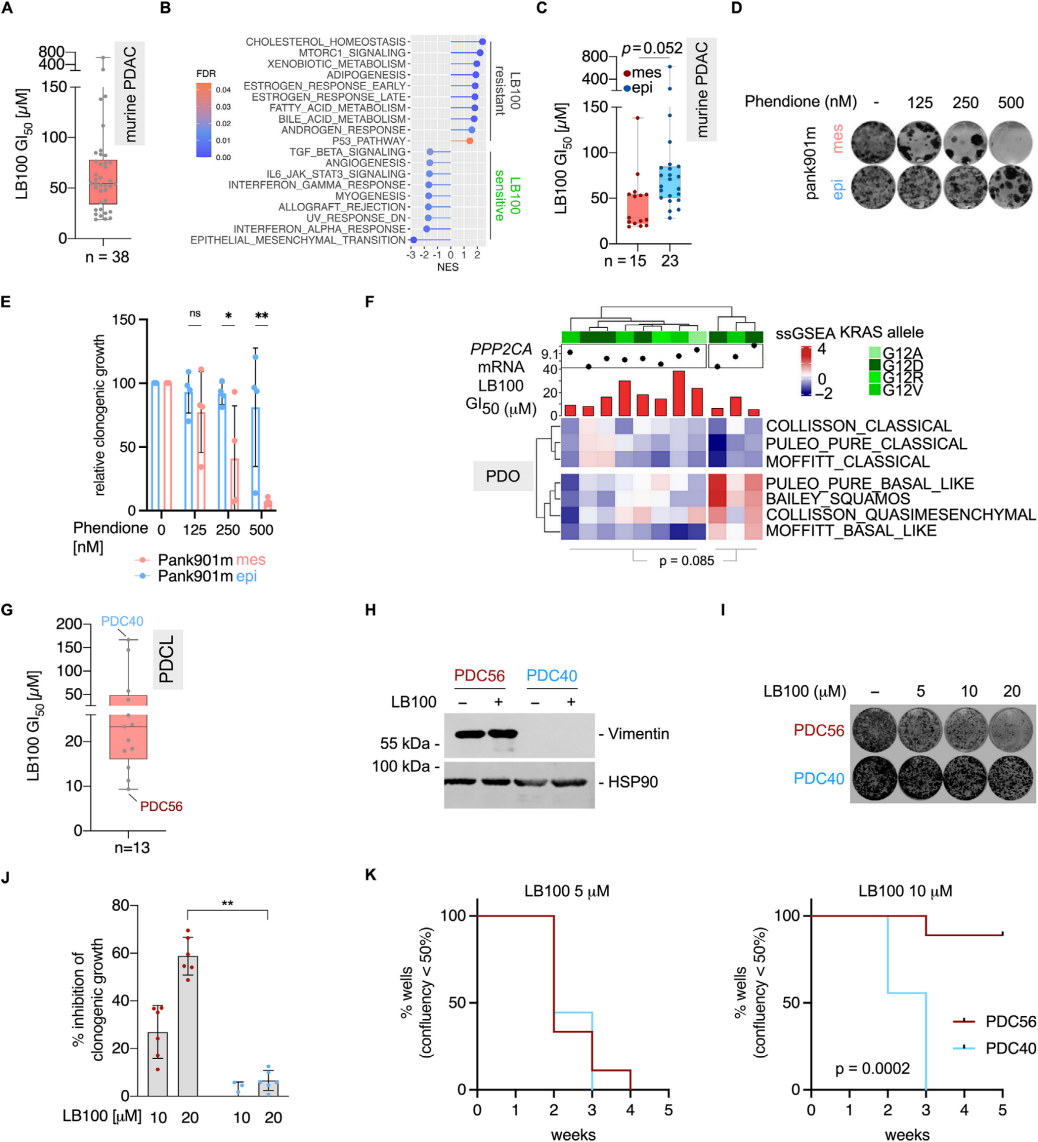
A mesenchymal lineage is PP2Ai sensitive. (A) Murine PDAC cells were treated with LB100 in a 7‐point dilution for 72 h and assayed for cell viability (*n* ≥ 3). Displayed are the half‐maximal growth inhibitory (GI_50_) concentrations (*n* = 38). (B) Depicted are the HALLMARK signatures from a preranked GSEA analysis. LB100 GI_50_ values from (A) were correlated with mRNA expression and the Pearson correlation coefficient was used as a rank for the GSEA. (C) LB100 GI_50_ values of murine epithelial (*n* = 23) and mesenchymal (*n* = 15) PDAC cell lines from (A) were compared. *p* Value of a Mann–Whitney test is depicted. (D) Displayed is one representative colony formation assay of the epithelial and mesenchymal subparts of the Pank901m cell line after 14 days treatment with the indicated concentrations of the PP2Ai phendione. (E) Quantification of four independent biological replicates from (D) (two‐way ANOVA, *p value < 0.05, **p < 0.01). (F) Displayed are the GI_50_ concentrations of patient‐derived PDAC organoids (human PDOs, *n* = 11) after treatment with LB100. GI_50_ values were calculated from at least two independent experiments. GI_50_ values are placed into the context of ssGSEA values of the indicated signatures, the *PPP2CA* mRNA expression values (normalized, log2 transformed), and the oncogenic *KRAS* allele. (G) Dose responses and viability were determined in human PDCL lines (*n* = 13) as described in (A) and the GI_50_ values were calculated. The most sensitive and most resistant line is depicted. (H) Western blot analysis of Vimentin after LB‐100 treatment for 6 h (10 µM) in the indicated PDCL lines. HSP90: loading control. One representative blot out of three experiments is displayed. (I) and (J) Inhibition of growth (%) was measured by a clonogenic growth assay of an LB‐100 sensitive (PDC56, red) and a resistant (PDC40, blue) PDAC–PDCL cell line after treatment with indicated concentrations of LB‐100 for 14 days. One representative image of the experiment is displayed (I), quantification of *n* ≥ 3 experiments (J). **Mann–Whitney test *p *< 0.01). (K) In situ resistance assay. Depicted are out‐growth curves for a mesenchymal (red, PDC56) and epithelial (blue, PDC40) patient‐derived cell line. Cells were treated with the indicated doses of LB100. All experiments were done in nine technical replicates and wells over 50% confluency were scored as out‐grown. Log‐rank test was used to compare the curves and the *p* value is depicted.

To identify markers that will allow for the precise use of PP2Ais in the clinic and to gain mechanistic insights into PP2A functions, we investigated a panel of well‐characterized murine Kras^G12D^‐driven PDAC cell lines [4] for PP2Ai sensitivity. We used LB100, a PP2Ai under clinical development [19]. The LB100 half‐maximal growth inhibitory concentration (GI_50_) was highly variable in such models and GI_50_ values in sensitive murine PDAC lines were around 20 µM (Figure 2A). GI_50_ values ranging from 0.4 to 20 µM have been reported in the literature [18]. This reported wide range might be due to the hydrolysis of LB100 [18].

Next, we correlated transcriptomic data to the GI_50_ values and used the correlation coefficient as a rank to perform a preranked gene set enrichment analysis (GSEA) as described [27] (Figure 2B). Interestingly, the signature for the epithelial‐to‐mesenchymal transition (EMT) was top‐scored and negatively correlated with the LB100 GI_50_ values, suggesting that mesenchymal murine PDAC cells are more LB100‐sensitive than epithelial ones. Consistent with the GSEA results, we observed lower LB100 GI_50_ values in mesenchymal PDAC lines (Figure 2C). In agreement herewith, LB100 induced higher clonogenic growth inhibition in the mesenchymal murine PDAC lines (Figure S1A). To further substantiate these findings, we used murine PDAC lines in which the epithelial and mesenchymal parts were separated by a differential trypsinization protocol. Since the genetic landscape of these models is similar [4, 28], they allow to associate drug sensitivity with cellular phenotypes, thereby reducing the influence of genetic confounders. Again, the mesenchymal cell lines were more LB100‐responsive than the epithelial ones (Figure S1B–G).

To corroborate our results, we employed another PP2Ai, namely 1,10‐phenanthroline‐5,6‐dione (phendione) [29]. In alignment with the former findings using differentially trypsinized cells, mesenchymal sublines exhibited a markedly heightened sensitivity to phendione (Figure 2D,E).

Additionally, we used patient‐derived cell lines (PDCL) and organoids (PDO) to cross‐species validate our data. In these translational models, we observed LB100‐sensitive and ‐resistant PDOs (Figure 2F). When the PDOs were stratified into classical and basal‐like subtypes, based on single‐sample GSEA analysis, the more basal‐like PDOs exhibited lower GI_50_ values (classical PDO GI_50_ 19.8 µM, basal‐like PDO GI_50_ 9.2 µM) (Figure 2F). In PDCLs, the line with the lowest GI_50_ value (PDC56) (Figure 2G) expresses vimentin, unlike the most resistant line (PDC40) (Figure 2H).

In addition to clonogenic growth assays, which demonstrated increased sensitivity of the vimentin‐expressing line (Figure 2I,J), we performed a comparative analysis of PDCL lines using an in situ resistance assay [30]. This assay investigates the outgrowth of cells under treatment over weeks. Here, growth of the vimentin‐expressing line PDC56 was markedly delayed under the 10 µM LB100 treatment (Figures 2K and S1H). Additionally, we assessed the association of LB100 sensitivity in a PDCL panel that was developed within the Clinical Research Unit 5002 (CRU5002), treating both mesenchymal and epithelial PDCLs with LB100 (Figure S1I). Consistent with previous findings, the GI_50_ values for LB100 were lower in mesenchymal PDCL lines (Figure S1I).

To better delineate the molecular determinants of LB100 responsiveness, we analyzed mesenchymal murine PDAC cells, which comprise two populations with differing sensitivity to LB100 (Figure 2C). We used GSEA to compare both populations and to find potential pathways connected to PP2Ai sensitivity. Interestingly, mRNA splicing and protumorigenic pathways, like the E2F and MYC transcription factor pathways, characterize the sensitive mesenchymal population (Figure S1J).

These data indicate that a mesenchymal PDAC cell lineage, characterized by activation of proproliferative pathways and the splicing machinery, exhibits increased sensitivity to LB100, highlighting a potential phenotype that could be utilized for patient stratification.

### 2.3 LB100 Treatment Activates Characteristic Stress Responses

**FIGURE 3:**
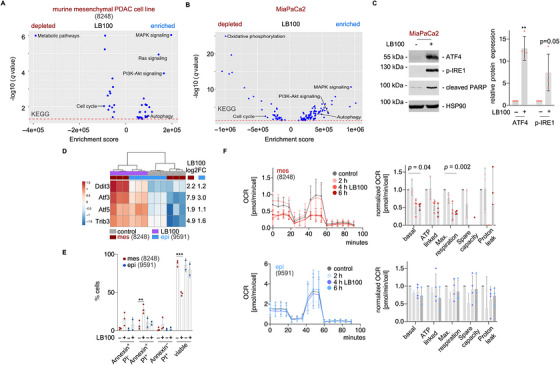
Characterization of the LB100‐induced cellular response. (A and B) Depicted are the KEGG signatures after GSEA analysis, including the enrichment score and the corresponding *q*‐value (−log_10_), of the mRNA expression profile of mesenchymal murine PDAC cells (A, 8248) or the human MiaPaCa2 cell line (B) after treatment with LB100 for 6 h with 20 or 10 µM, respectively. (C) *Left*: Displayed are the ATF4, p‐IRE1, and cleaved PARP blots after treatment of the mesenchymal human MiaPaCa2 cell line with LB100 (10 µM, 6 h). One representative blot out of four independent biological experiments is shown. HSP90: loading control. *Right*: Quantification of the relative protein expression for ATF4 and p‐IRE1 (*n* = 4, paired two‐tailed *t*‐test **p < 0.01, or indicated). (D) Heatmap representing the mRNA expression of ATF4‐target genes in these mesenchymal and epithelial lines after 2 h of treatment with LB100 (*n* = 3) determined by iRNA‐seq. *Right*: Log2FC values of the genes. (E) Displayed are the percentage of viable (Annexin V‐/Pi‐), early apoptotic (Annexin+/Pi‐), late apoptotic (Annexin V+/Pi+), or necrotic (Annexin V‐/Pi+) cells, which were measured by Annexin/PI staining after 6 h of treatment with LB100 or vehicle control. (mesenchymal, 8248: *n* = 3, epithelial, 9591 *n* = 2, **p < 0.01, ***p < 0.001, two‐tailed paired *t*‐test). (F) A mesenchymal cell line (8248, upper panel, red) and an epithelial PDAC cell line (9591, lower panel, blue) was treated with 20 µM LB100 for 2, 4, and 6 h or vehicle control. Oxygen consumption rate (OCR) was measured with a Mito Stress Seahorse Assay and used to calculate mitochondrial respiration values (*p* values of an ANOVA with Bonferroni correction are indicated (*n* ≥ 3)). Each dot represents one biological replicate. Bar graphs: Quantification of the OCR values from four independent experiments.

LB100 paradoxically activates oncogenic signaling pathways, triggering a stress response that is detectable by increased phosphorylation of the unfolded protein response (UPR) orchestrator inositol‐requiring enzyme 1 (IRE1) [31]. Consistently, GSEA of LB100‐treated mesenchymal PDAC cells revealed significant enrichment of signatures associated with oncogenic pathway activation, including KRAS, MAPK, and PI3K signaling (Figure 3A,B and Table S2). Furthermore, treatment of human mesenchymal PDAC cells with PP2Ai led to increased phosphorylation of IRE1 and elevated expression of activating transcription factor (ATF)4, a master regulator of the endoplasmatic reticulum (ER)/integrated stress response (ISR) (Figure 3C). Comparative analysis of ATF4 target gene expression in murine PDAC cells demonstrated its induction, which was more pronounced in mesenchymal compared with epithelial murine PDAC cells (Figure 3D). Activation of the stress response was associated with programmed cell death induction in both human and murine mesenchymal PDAC cells (Figure 3C,E).

ATF4 induction, mediated by eIF2α phosphorylation, is associated with translational stalling and stress granule (SG) formation [32, 33]. Consistent with ATF4 induction in mesenchymal PDAC cells, we detected elevated eIF2α phosphorylation in human PDCL (Figure S2A) and subsequent SG formation, as evidenced by cytoplasmic granules positive for the canonical SG marker TIA‐1 (Figure S2B). The positive control H_2_O_2_ significantly increased the number of SGs in mesenchymal murine PDAC cells comparable to LB100 treatment (Figure S2B).

Beyond these stress responses, our GSEA analysis revealed concomitant suppression of metabolic pathways and activation of autophagy‐related gene signatures (Figure 3A,B). Therefore, we measured live‐cell metabolism using Seahorse XF assay to determine the activity of the respiratory chain. LB100 treatment significantly and selectively reduced oxidative phosphorylation in mesenchymal PDAC cells (Figure 3F).

Autophagy signatures are enriched in LB100‐treated PDAC cells (Figure 3A,B). This lysosome‐mediated limited self‐eating process is a homeostatic system that is linked to the ISR [34]. Immunoblotting for the vesicle‐forming autophagy marker phosphatidylethanolamine‐conjugated LC3 (LC3‐II) revealed a significant increase in LC3‐II upon the treatment with LB100, selectively in mesenchymal PDAC cells (Figure S2C). In addition, we determined the lysosomal content using LysoTracker. LB100 treatment increased the LysoTracker signal exclusively in sensitive human and murine mesenchymal PDAC cells (Figure S2D–G). The energy sensor and autophagy inducer AMP‐activated protein kinase (AMPK) controls autophagy by direct phosphorylation of the apical autophagy regulator Unc‐51‐like autophagy activating kinase 1 (ULK1) [35]. LB100‐induced phosphorylation of AMPK at T172 was detected in both cellular phenotypes (Figure S2H). However, decreased phosphorylation of S751 of ULK1 was selectively observed in mesenchymal cells. Phosphorylation of ULK1 at this phospho‐site is conducted by mTOR and prevents activation of ULK1 and interaction of ULK1 with AMPK [36].

To this end, our analysis reveals that LB100 induces a cellular phenotype characterized by ER‐stress/ISR activation, concurrent metabolomic reprogramming, and subsequent cell death.

### 2.4 CRISPR–Cas9 Screen Identifies Executioner Nodes Triggered by LB100

**FIGURE 4:**
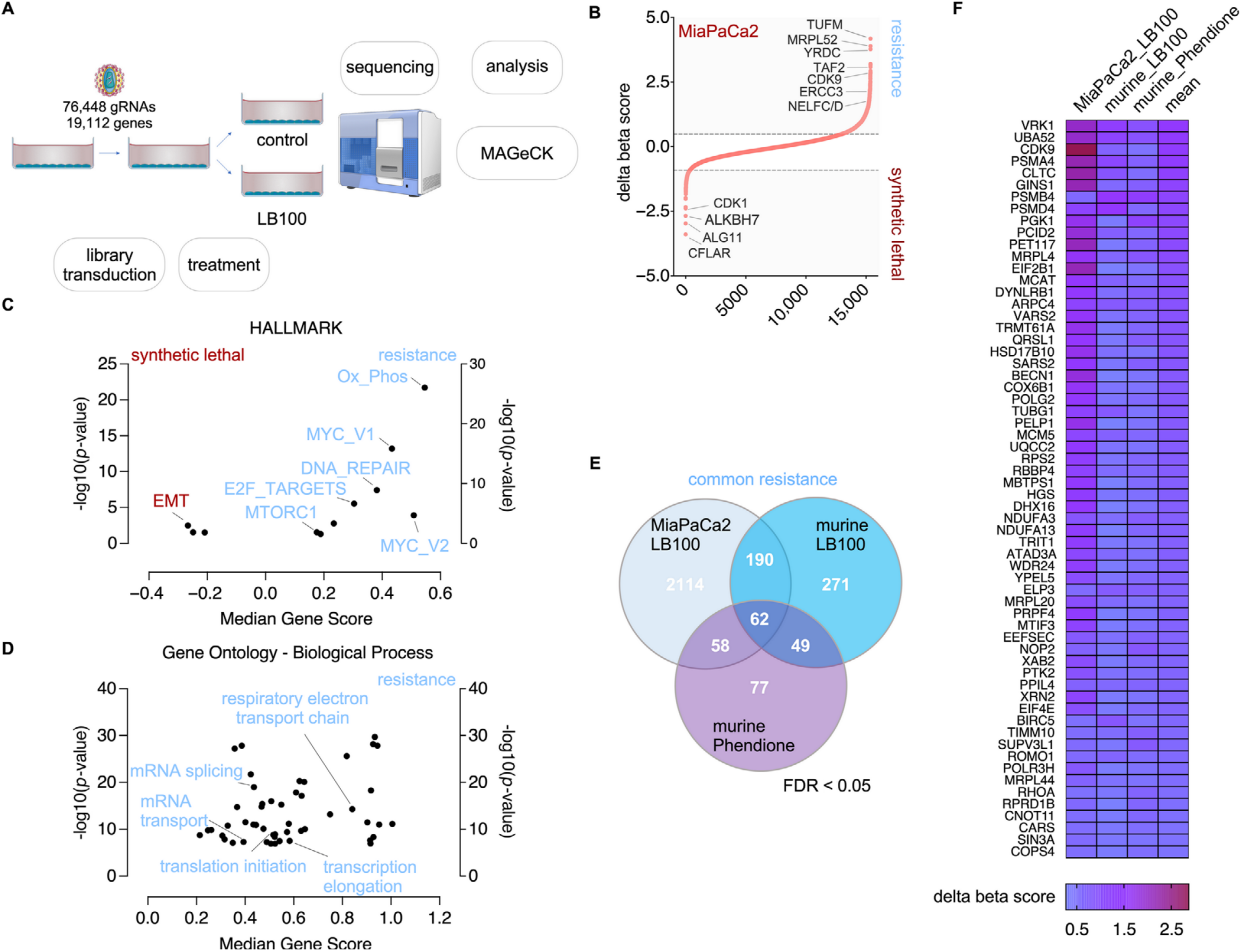
A CRISPR–Cas9 screen identifies LB100‐triggered nodes. (A) Schematic representation of the CRISPR–Cas9 dropout screen and analysis. (B) Distribution of the calculated delta beta scores of the drop‐out screen in MiaPaCa2 cells. Resistance: positive beta score, blue or synthetic lethal: negative beta score, red. Dashed lines: above or below FDR < 0.05. (C) and (D) Delta beta scores of the MiaPaCa2 screen were used as a rank for a preranked GSEA using the GeneTRAIL3 web interface and the HALLMARK (C) or GO BP signatures (D). Blue: LB100 resistance, red: synthetic lethality. (E) Venn analysis of the drop‐out screen in MiaPaca2 cells (with LB100), and murine PDAC cell line 9091 (with LB100 or Phendione). (F) Color‐coded delta beta scores of the 62 common genes, whose targeting confers a survival advantage under PP2A inhibition. Genes were ranked by the mean delta beta score of all screens.

To decode the complex cellular response of mesenchymal PDAC cells to PP2A inhibition and to unbiasedly determine the genetic underpinnings of the cellular susceptibility to LB100, we performed a genome‐wide CRISPR–Cas9 knock‐out screen (Figure 4A). MiaPaCa2 Cas9 expressing cells were transduced with the Brunello sgRNA library containing 76,448 gRNAs targeting 19,112 genes [37] and were treated with LB100 (GI_30_ = 5 µM) for 14 days. The sgRNA‐mediated effects on single genes (beta‐scores) were determined with the MAGeCK pipeline [38]. Delta‐beta scores were calculated, and we considered genes with an FDR < 0.05 as relevant (Figure 4B and Table S1). Pathway analyses of genes whose knock‐out confers a survival advantage in LB100‐treated MiaPaCa2 cells were centered around proliferation, DNA repair, transcription, mRNA metabolism, splicing, translation, and mitochondrial biology (Figure 4C,D). Genes that are potentially synthetic lethal with LB100 treatment were connected to EMT (Figure 4C and Table S1). No significantly enriched synthetic lethal pathways were detected in the gene ontology (GO) analysis (Figure 4D and Table S1).

To cross‐species and cross‐inhibitor validate the screening results, we used a murine mesenchymal PDAC cell line and the PP2Ai phendione [29] in the framework of a genome‐wide drop‐out screen with the murine Brie library, covering 19,674 genes with 78,637 gRNAs (Figure S3A,C and Table S1) [37]. Once again, we observed that genes involved in chromatin and DNA biology, mRNA regulation, translation, and metabolism confer resistance to PP2A inhibition (Figure S3B,D and Table S1). In all screens, we detected an overlap of 62 genes whose CRISPR targeting conferred a survival advantage in PP2Ai‐treated cells (Figure 4E,F).

These genetic screening experiments uncover that genes involved in transcription, DNA repair, mRNA regulation, translation, and metabolism play key roles in modulating the response to PP2A inhibition.

### 2.5 PPP2R1A Knockdown Adaptation Phenocopies the LB100 Resistance Pathway

To further support our analysis of the PP2Ai response, we knocked down the scaffolding PP2A unit *PPP2R1A* using CasRx in MiaPaCa2 cells [39]. We chose PPP2R1A because it is a key PP2A scaffold subunit and its siRNA‐mediated knockdown has been used to define the PP2A‐regulated phospho‐proteome [40]. We verified the knockdown of *PPP2R1A* by western blotting (Figure S4A,B). *PPP2R1A*‐depleted cells showed no significant change in proliferation (Figure S4C). Given that genetic screens indicate a pronounced fitness defect upon *PPP2R1A* knockout in MiaPaCa2 cells (Figure S4D), we interpret the absence of a growth phenotype after *PPP2R1A* knockdown as suggestive of compensatory adaptive processes. To further characterize the model system, we profiled transcriptomes. Analysis of mRNA samples collected over a 3‐week period revealed that the *PPP2R1A* knockdown was reduced at the latest time point analyzed (Figure S4E). Therefore, we conducted the analysis with the two samples with a distinct *PPP2R1A* knockdown, excluding the third sample in the higher passage. Here, the transcriptomes of *PPP2R1A* knockdown MiaPaCa2 cells aligned with the resistance‐associated pathways identified in the CRISPR screens, including metabolism, transcription, translation, and splicing (Figure S4F,G). This suggests that the LB100 CRISPR screen captures the on‐target biology of PP2A inhibition, as it elicits the similar compensatory programs observed upon *PPP2R1A* knockdown. Furthermore, *PPP2R1A* knockdown MiaPaCa‐2 cells retained some of the pathway changes observed after short‐term LB100 treatment, including oxidative phosphorylation (Figure S4H).

Therefore, the PPP2R1A knockdown prompted compensatory transcriptomic changes that mirrored the resistance pathways uncovered by the LB100 genetic screen, suggesting these effects result from on‐target PP2A inhibition.

### 2.6 LB100 Treatment Affects Splicing and Transcriptional Elongation

To prioritize the orthogonal validation experiments, we used the PDAC Clinical Proteomic Tumor Analysis Consortium (CPTAC) dataset [41]. Analysis of ssGSEA scores [42] revealed that the human PDAC subtype with high EMT signature scores clusters with signatures reflecting enhanced KRAS signaling, hypoxia, or glycolysis (Figure S5A). PDACs with a high EMT signature score have a worse prognosis (Figure S5B) and enrich signatures that are associated with transcription and RNA polymerase II (RNA Pol II) (Figure S5C), which correlates both processes to the EMT phenotype.

To identify PP2A‐associated biological processes, we analyzed phosphorylation events that exhibited a negative correlation with PPP2CA expression. An overrepresentation analysis of negatively correlated phosphoproteins revealed only four significant GO signatures, linked to transcription, transcriptional elongation, and mRNA metabolism (Figure S5D). Furthermore, PPP2CA protein expression was negatively correlated with phosphorylation of relevant transcriptional regulators (Figure S5E).

**FIGURE 5:**
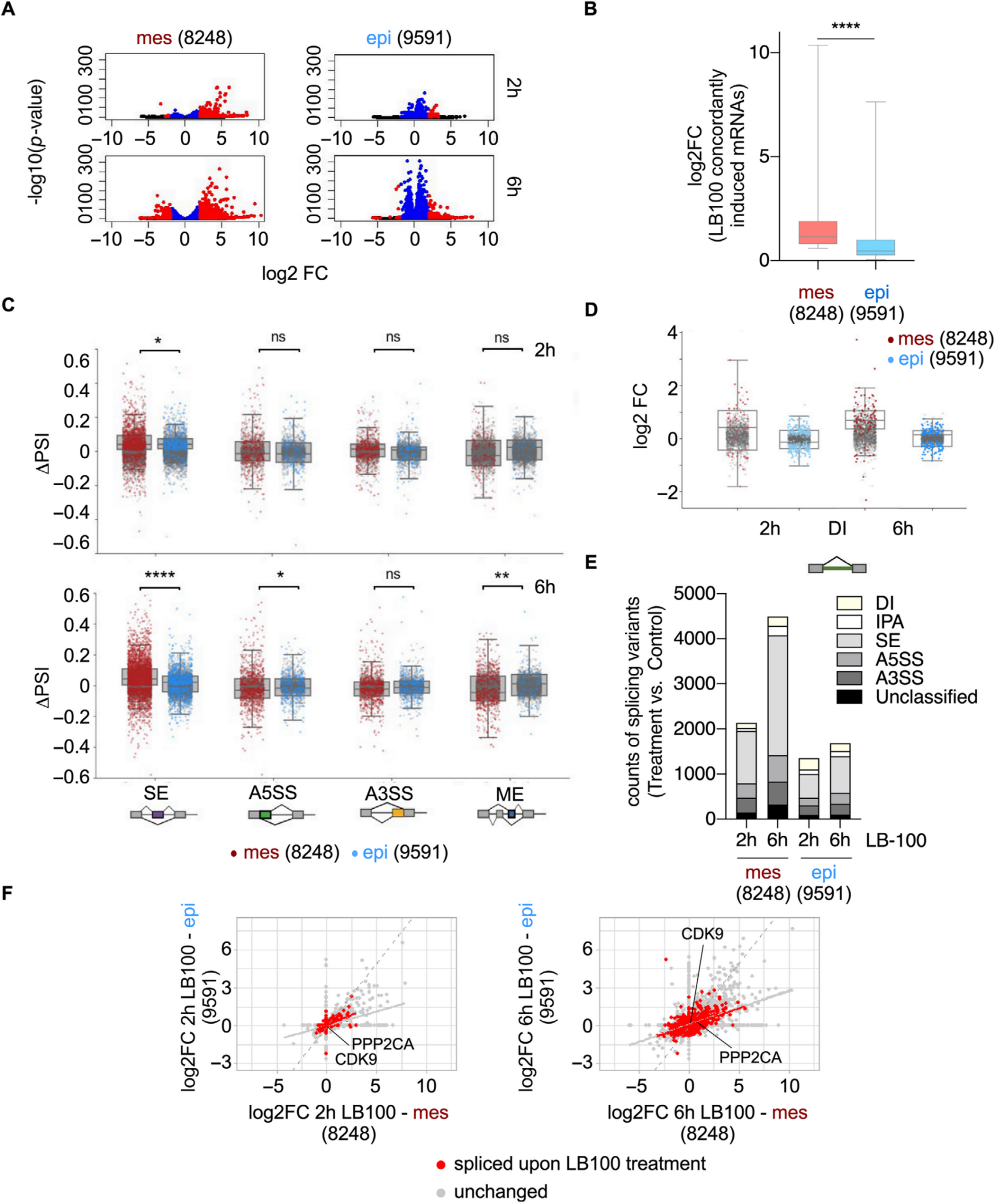
Induction of splicing upon LB100 treatment. (A) mRNA expression analysis of a mesenchymal (8248) and epithelial (9591) murine cell line after 2 and 6 h of LB100 treatment (20 µM). Displayed are the log_2_ fold changes of all regulated genes and their corresponding *p* value (−log10). Blue: *p* value < 0.05, red: *p* value < 0.05 and log2FC > 2. (B) Displayed is the Log2FC of all concordantly in epithelial and mesenchymal lines induced mRNAs from (a) after 6 h of treatment with LB100 (*n* = 1288, two‐tailed unpaired *t*‐test ****p value < 0.0001). (C) Deep RNA sequencing was performed for splicing variants analysis in a mesenchymal (8248) and an epithelial (9591) murine PDAC cell line after LB100 treatment (20 µM) for 2 and 6 h. The distribution of change in percentage spliced in (delta PSI) between the mesenchymal (red) and epithelial (blue) cell line after 2 h (top) and 6 h (bottom) of LB100 treatment. Vertical lines within boxes denote median, edges of boxes the first and fourth quartiles, and whiskers the minimum/maximum values. The difference in the distributions between two cell lines is tested using KS test (*p < 0.05, ****p < 0.0001). SE = skipped exons, A5SS = alternative 5′ splicing site, A3SS = alternative 3′splicing site, ME = mutually exclusive exon. (D) Detailed Log2FC of detained intron changes after 2 and 6 h of treatment. Colored are the changes with an average count over 100 either in the control or treatment with a *p* value under 0.05. DI = detained introns. (E) Displayed are the counts of the analyzed splicing variants (treatment vs. control) from (C) and (D) for each time point in the mesenchymal (8248) and epithelial (9591) cell line, IPA = intronic polyadenylation. (F) Comparison of gene expression (gray, log2FC, results from a) and corresponding splicing changes in the genes (red, results from (c) and (d)) between mesenchymal and epithelial cell lines after 2 h (left side) and 6 h (right side) of treatment. Dashed gray line: hypothetical linear regression of gene expression, straight line: actual gene regression line of splicing affected genes. CDK9 and PPP2CA gene expression is highlighted.

Based on these findings, we explored the effects of LB100 on mRNA splicing and transcriptional regulation. To investigate the effects of LB100 on the transcriptome, we determined the regulation of mRNA using RNA deep sequencing in murine PDAC cells. We observed that LB100 treatment predominantly led to the upregulation of mRNA transcription. Overall, the induction of mRNA over time is more pronounced in mesenchymal PDAC cells. These effects were already evident 2 h after the treatment with LB100 (Figure 5A and Table S2), excluding secondary or cell death‐related mechanisms. The relative extent of concordantly LB100‐induced mRNAs was higher in mesenchymal than in epithelial cells (Figure 5B).

**FIGURE 6:**
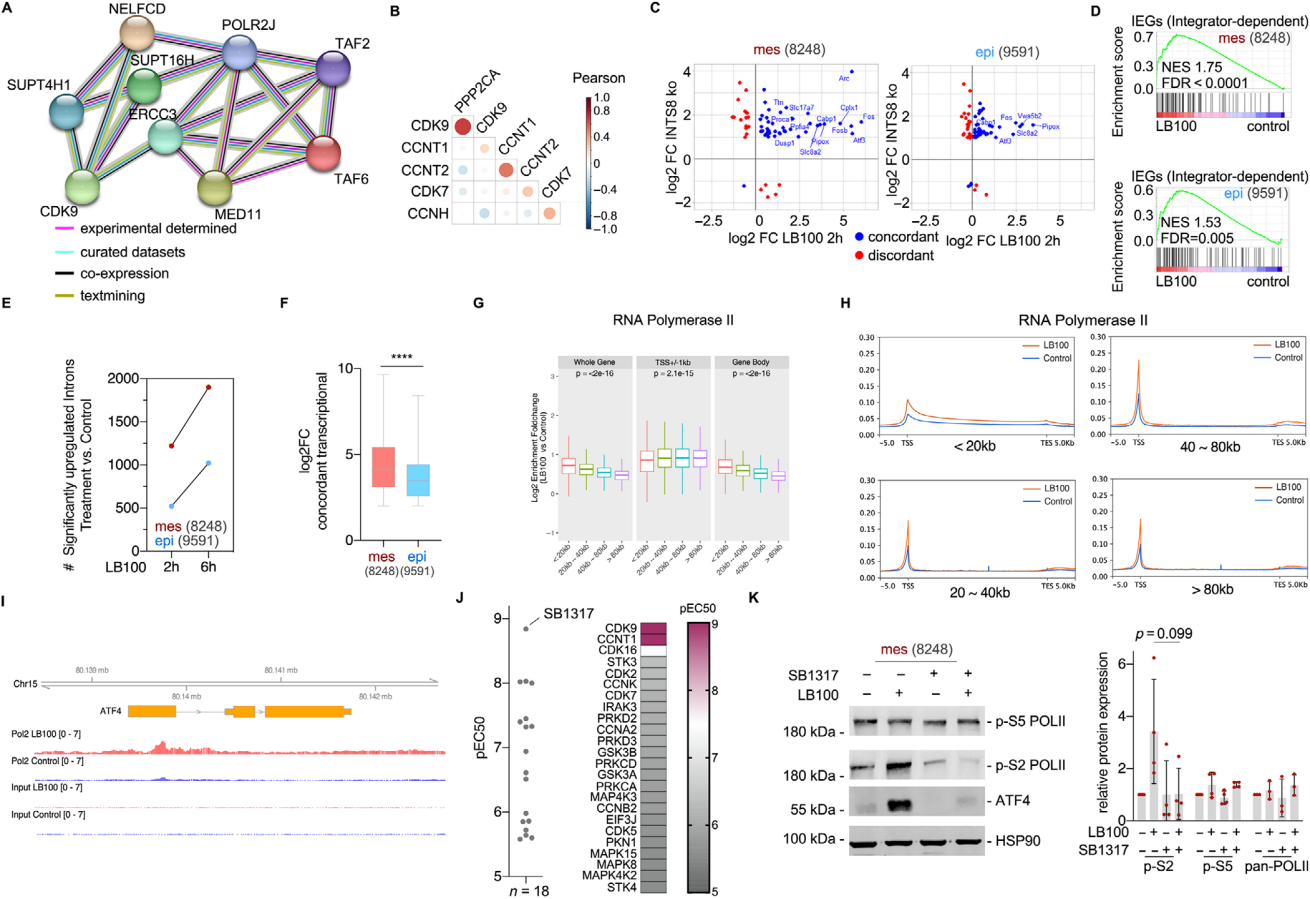
LB100 treatment activates transcriptional activation. (A) Screening hits in MiaPaCa2 cells with a delta beta‐score > 1.5 were manually annotated for transcriptional regulation and control of RNA Pol II. Hits were analyzed with the STRING platform (medium confidence 0.7). (B) *PPP2CA* mRNA expression of murine PDAC cell lines (*n* = 38) was correlated with indicated mRNAs. The Pearson correlation coefficient is color coded. (C) RNA sequencing data were compared with sequencing data after INTS8 knockdown in HEK293 cells. Displayed are the significantly overlapping concordantly (blue) and discordantly (red) regulated genes. (D) Fold induction of genes defined by iRNA‐seq upon 2 h of treatment with LB100 from were used as a rank for preranked GSEA with a gene‐set of IEGs activated by EGF integrator dependent. (E) iRNA‐seq analysis was done to determine intron and exon expression (*p* < 0.05 and log2FC > 2) in the mesenchymal (8248) and epithelial (9591) murine cell line after 2 and 6 h of treatment with LB100 (20 µM) compared with vehicle control. (F) Log2 FC of introns induced upon LB100 treatment from (E) were compared. ****p < 0.0001 (unpaired *t*‐test, *n* = 556). (G) Fold enrichment of RNA Pol II enrichment across indicated gene regions following 2 h of LB100 (5 µM) treatment in mesenchymal 8248 cells. Enrichment was calculated for the indicated gene length groups. (H) RNA Pol II coverages across the whole gene of the indicated different gene lengths with and without 2 h LB100 treatment in mesenchymal 8248 cells. (I) Peak distributions RNA Pol II across the ATF4 gene region in murine 8248 cells with and without 2 h of LB100 treatment. (J) SB1317 CDK9 inhibitor selectivity profile based on kino‐bead assays and accessed via https://www.proteomicsdb.org/. Displayed are the pEC50 values for SB1317 in comparison with all other CDK9 inhibitors of the database (left) and the color‐coded pEC50 for all targets of SB1317 (right). (K) *Left*: Western blot analysis of the murine mesenchymal (8248) cell lines after treatment with LB100 (20 µM, 6 h), SB1317 (0.5 µM, 6 h) or a combination of both. Western blots demonstrating phosphorylation of serine 5 and 2 of RNA Pol II and ATF4. HSP90 serves as a loading control. All experiments were done at least in triplicates, one representative blot is shown. Same lysate was blotted on different membranes; each quantification is done to the corresponding loading control of the membrane. *Right*: Quantification of RNA Pol II phospho‐sites in the mesenchymal cell line 8248 (ANOVA with Tukey's correction, *p* value indicated or *p < 0.05, *n* ≥ 3).

To investigate an impact on splicing events, we used our paired‐end deep RNA sequencing (RNA‐seq) after 2 and 6 h of treatment with LB100. Here, we analyzed splicing events of skipped exons (SE), 5′ or 3′ prime splice sites (A5SS or A3SS) or mutually exclusive exons (ME) using the distribution of change in percentage spliced in (delta PSI) between 8248 (mesenchymal, red) and 9591 (epithelial, blue) murine cells after LB100 treatment (Figure 5C). For determining the changes in detained introns (DI) between the two subtypes, use was made of their fold change between untreated and treated samples (Figure 5D). Mesenchymal cells showed overall 2.5 times increased splicing changes after 6 h of LB100 treatment, compared with epithelial cells (Figure 5C–E). Of all splicing changes, SE were detected as the most affected and regulated form, followed by A5SS or A3SS. Alternative polyadenylation and DI were affected less frequently (Figure 5C–E). To uncover the splicing changes effect on the transcriptional output, an overlay of all splicing changes with changes in gene transcription was made (Figure 5F). In epithelial and mesenchymal PDAC cells LB100‐treatment induced splicing, however genes affected by splicing changes seem not to be responsible for the significant major change in gene expression (Figure 5F). While PP2A has been linked to splicing regulation [43], de‐phosphorylation of splicing regulators was connected to the phosphatase [40, 43], and LB100‐induced splicing changes in PDAC cells mirror findings in colorectal cancer  [43], the cellular response in the mesenchymal PDAC lineage could stem from enhanced transcriptional out‐put secondary leading to increased splicing events. Supporting this consideration, our genetic screens demonstrate that transcriptional regulators affect PP2Ai sensitivity significantly (Figure 4). In MiaPaCa2 cells, the knock‐out of transcription cycle nodes (like CDK9), components of the basal transcription machinery (like TAF2), basal transcription factor II (TFIIH) components (like ERCC3), or a subunit of RNA Pol II (POLR2J) (Figure 6A) confers a survival advantage and CDK9 was a consistent hit in all genetic screens (Figure 4F). Furthermore, we observed a correlation of *Ppp2ac* mRNA to the *Cdk9* mRNA (Pearson *r* = 0.68, *p* = 3.3 e−06) in murine PDAC cells (Figure 6B).

A noncanonical PP2A complex lacking regulatory B subunits was described [44, 45]. PP2A is part of the integrator multiprotein complex, which regulates RNA Pol II promoter proximal pausing and elongation [46, 47]. Integrator subunit 8 (IntS8) interacts with PP2A to recruit PP2A to the complex and chromatin resulting in dephosphorylation of the C‐terminal domain (CTD) of RNA Pol II [48]. This is in line with the note that the transcription cycle [49] relies on phosphatase activity [50] and in consequence, the integrator–PP2A complex leads to gene repression by antagonizing transcriptional elongation driven by CDK9 [44, 45, 48, 51]. To explore the relationship between the LB100 response and integrator‐controlled gene expression, we compared transcriptomic changes following *INTS8* knockout in HEK293T cells (GSE150844) [48] with our data on PP2A inhibition in murine PDAC cells. Interestingly, we detected an overlapping INTS8‐controlled and LB100‐induced mRNA expression network in mesenchymal as well as epithelial PDAC cells (Figure 6C). This network included immediate early genes (IEGs), like the AP1 family transcription factors FOS and FOSB or the ATF family member ATF3 (Figure 6C). To further corroborate these findings, we performed GSEA analysis of our iRNA‐seq data (Table S3) using a gene set of integrator‐dependent, EGF‐induced IEGs [52] and observed a significant enrichment in LB100‐treated PDAC cells (Figure 6D).

An additional analysis of the human *INTS8*‐knock‐out HEK293T data (GSE150844) and RNA‐seq data from LB100‐treated MiaPaCa2 cells showed mostly concordant gene expression changes across both datasets. This involves IEGs from the AP‐1 and ATF transcription factor families (Figure S5F). Consistently, the IEGs induced by EGF [52] were notably enriched in LB100‐treated MiaPaCa2 cells (Figure S5G).

To measure activation of the transcriptional machinery, we applied iRNA‐seq, a computational method measuring transcriptional activity by intron coverage from RNA‐seq data. iRNA‐seq gave similar results as global run‐on sequencing or RNA Pol II chromatin immunoprecipitation (ChIP‐seq) [53]. The number of significantly upregulated introns was distinctly higher in mesenchymal PDAC cells compared with epithelial PDAC cells (Figure 6E and Table S3). Increased induction of introns is already evident after 2 h and was further pronounced after 6 h (Figure 6E). Furthermore, the qualitative induction, illustrated as log2 fold change, is higher in mesenchymal cells (Figure 6F). Together, this points to higher transcriptional output in the mesenchymal cell line investigated upon LB100 treatment (Table S3).

To confirm this, we performed ChIP‐seq in mesenchymal murine PDAC cells to analyze RNA Pol II binding. We observed enrichment of RNA Pol II binding, particularly at shorter genes (<20 kb) (Figure 6G,H). Consistently, we found that LB100 primarily induced expression of shorter genes, with a negative correlation between transcript length and induction magnitude (Figure S6A,B). RNA Pol II binding is exemplified at key UPR genes, including ATF4, HSPA5, EIF2A, and ATF6 (Figures 6I and S6C). These findings further connect the LB100 response to integrator, as integrator inactivation releases RNA Pol II into elongation—preferentially transcribing short genes while failing to transition into the fully productive state required for long gene transcription [54, 55, 56].

To substantiate that the antagonism of CDK9 to PP2A is imbalanced upon treatment with LB100, we used the CDK9 inhibitor SB1317. This agent demonstrated the lowest half‐maximal effective concentration of all CDK9 inhibitors accessible via the ProteomicsDB web interface (https://www.proteomicsdb.org/) (Figure 6J). Consistent with our hypothesis, that LB100 induces a CDK9‐dependent process, we detected induction of RNA Pol II serine 2 phosphorylation in murine mesenchymal PDAC cells (Figure 6K). SB1317 reduced the LB100‐induced phosphorylation of RNA Pol II at serine 2 (Figure 6K). Since we observed ATF4 induction upon LB100 treatment, we used ATF4 as a surrogate marker of the LB100‐induced stress. The LB100‐mediated induction of ATF4 is reduced upon SB1317 cotreatment (Figure 6K).

These observations indicate that the program activated by PP2A inhibition is linked to CDK9.

## 3 Discussion

We show here that PP2A is a therapeutic target in aggressive mesenchymal PDACs and provide evidence that PP2A inhibition activates and shifts RNA Pol II toward elongation, which was linked to the induction of ER‐stress/ISR regulator ATF4. Our findings corroborate recent reports that PP2A is an important therapeutic target in PDAC and demonstrate that cells become particularly dependent on its catalytic subunit PPP2CA when the paralog PPP2CB is lost or MYC is amplified [57].

A more aggressive mesenchymal group of the disease was recently described by us [4, 58] and other groups [6] and the EMT trajectory is an important component of inter‐ and intratumoral heterogeneity [7, 59, 60, 61]. In addition to pathways such as glycolysis, which are known to be active in basal‐like/mesenchymal PDACs, our work identifies an enrichment of transcription‐ and RNA Pol II‐associated signatures in this subtype. Moreover, the mesenchymal lineage drives PDAC evolution and progression, and depleting cells with mesenchymal features produced therapeutic efficacy in murine PDAC models [62]. These significant findings highlight the need for therapies targeting the mesenchymal lineage and support the continuous development of PP2Ai for PDAC treatment.

The multiprotein integrator complex is an important regulator of RNA Pol II [47, 63]. In addition to its other activities, such as processing small nuclear RNAs or noncoding RNAs and termination of transcription, the integrator complex is involved in pausing RNA Pol II downstream of the transcription initiation site and the control of elongation [46]. A complex of integrator with PP2A, called INTAC, was identified [44]. Integrator recruits PP2A to chromatin and directs the activity of the phosphatase to the heptad repeats of the CTD of RNA Pol II. This process reduces the transcription of protein‐coding genes [44]. Furthermore, integrator forms a complex with the pausing factors DSIF and NELF, which prevents the recruitment of the elongation factors SPT6 and PAF1 to RNA Pol II [45]. The interaction with PP2A is necessary for integrator‐dependent gene repression, mediated by dephosphorylation of CTD of RNA Pol II or associated factors, like SPT5 or NELFA [48, 54, 64, 65]. A recent study [51] showed that the integrator–PP2A complex antagonized CDK9‐driven transcriptional elongation and importantly demonstrated that the balance between CDK9 and the integrator–PP2A complex could be addressed therapeutically [49, 51]. In leukemic and solid cancer models, the therapeutic synergy between inhibition of CDK9 with activators of PP2A was demonstrated and resulted in enhanced RNA Pol II pausing and augmented cell death [51]. This PP2A–CDK9 antagonism is also described in the context of PDAC. The PP2A inhibitory nuclear oncoprotein SET acts in concert with CDK9 to maintain RNA Pol II CTD phosphorylation and active transcription [66]. Our data extend these observations by showing that also PP2Ais impinge on the CDK9–PP2A balance and we provide evidence for a survival advantage of CDK9 targeted cells under LB100 treatment in our CRISPR–Cas9 genetic screening experiment. Consistently, we detect increased binding of RNA Pol II and phosphorylation of RNA Pol II CTD upon PP2A inhibition. Furthermore, we find an overlap of a transcriptional program known to be repressed by integrator–PP2A, including IEGs [48, 64], to be activated in the used PDAC models upon PP2A inhibition. These data support the hypothesis that the integrator–PP2A/CDK9 antagonism shifts toward increased elongation upon treatment with LB100, with a preferential activation of short genes. Moreover, the data presented exhibit consistency with the significant upregulation of RNA Pol II phosphorylation at most transcription units, accompanied by enhanced elongation observed upon the induced degradation of INTS8, which prevents recruitment of PP2A to integrator [67]. Therefore, in contrast to the profound block of transcriptional elongation induced by CDK9 inhibition and PP2A activation, our data provide evidence that the unconventional inactivation of the elongation checkpoint is linked to a cellular stress response and can be exploited to tailor therapies.

Importantly our work demonstrates that mesenchymal PDAC cells are more susceptible to PP2A inhibition, enabling stratification of LB100 applications. We observed a correlation of PP2A expression with CDK9 in murine PDAC cells and PP2A expression is negatively correlated with phosphorylation of important transcriptional regulators in a human PDAC dataset. Mesenchymal PDAC cells are characterized by an increased gene dose of mutant KRAS [4, 68], a genomic feature linked to worse survival [10, 68]. Notably, the integrator complex has an important role in connecting ERK signaling to the productive transcription of IEGs [52]. Furthermore, the ERK kinase, a downstream effector of canonical KRAS signaling, was shown to phosphorylate NELFA with subsequent dissociation from RNA Pol II and transcriptional elongation of IEGs [64]. Since PP2A counteracts this process, higher ERK signaling output might shift the balance to efficient transcriptional elongation in the context of PP2A inhibition in mesenchymal PDAC cells. Whether the increased transcriptional output is connected to the accumulation of cytotoxic protein aggregates in mesenchymal PDAC cells treated with phendione remains to be determined [69].

Our data are in line with observations made in colon cancer models describing the paradoxical activation of oncogenic pathways upon PP2A inhibition [31, 70]. Treatment with LB100 induces the activation of ERK‐, JUN‐, and WNT/β‐catenin pathways, with increased phosphorylation of IRE1α, indicating an UPR [31]. Complementary CRISPR activation and CRISPR‐KO screens in colon cancer cells revealed that oncogenes like MAP3K1, CTNNB1, or MYC modulate the LB100 response [31]. Importantly, CRISPR‐KO screening experiments conducted in SW480 colon cancer cells revealed that knock‐out of components of the Mediator complex (e.g. MED12 or MED15), transcription factors (e.g. LEF1, CTNNB1, BCL9L, or EGR4), or the histone acetyltransferase p300 attenuate the LB100 response, an observation that aligns with our own data [31]. Furthermore, the integrator complex has recently been linked to the UPR. Following integrator depletion, RNA Pol II elongates into gene bodies but exhibits increased premature termination. This results in incomplete pre‐mRNAs containing retained introns. Retroelements within these intronic regions form double‐stranded RNAs, which activate protein kinase R and subsequently trigger the ER stress response [56].

### 3.1 Limitations of Our Study

Most of our data were generated using the small‐molecule PP2Ai LB100, chosen because it is in clinical development [19]. Although we validated findings with the structurally different PP2Ai phendione [29] and used a knock‐down of *PPP2R1A*, we cannot exclude the possibility that off‐target effects of LB100 contribute to the observed mechanisms and phenotypes. Furthermore, given PP2A's pleiotropic target spectrum, we cannot exclude the possibility that integrator‐, transcription‐, and CDK9‐independent mechanisms or other canonical PP2A complexes contribute to the observed phenotypes.

It likewise remains unclear why the transcriptional output of mesenchymal cells is different. Future experiments should rationally consider (i) the increased KRAS gene dosage [4, 10, 68, 71] in mesenchymal PDACs, and (ii) pathways being active in this subtype, such as glycolysis and inflammation, as both pathways have been linked to hyper‐transcription [72], a global increase in RNA output. However, additional mechanisms may be involved and warrant further investigation.

## 4 Conclusion

Considering the emerging paradigm that paradoxical activation of oncogenic processes may represent a novel strategy for cancer therapy [31, 70], our findings highlight transcriptional elongation activation as a potential mechanism within this framework. Additionally, our data provide a rationale for developing PP2A‐based combination therapies, a critical step toward translating PP2Ai into clinical use.

## 5 Material and Methods

Detailed material and methods can be found in Supporting Information.

### 5.1 Cell Lines

Murine pancreatic cancer cell lines were established from Kras^G12D^‐driven mouse models as described [4, 22, 73]. The cell lines were cultivated in high glucose DMEM medium (#D5796; Sigma–Aldrich) with 10% (v/v) fetal calf serum (FCS; #TMS‐013‐B; Merck Millipore, Berlin, Germany) and 1% (v/v) penicillin/streptomycin (#15140122; Life Technologies, Darmstadt, Germany). For splitting, the cells were washed with 1× Dulbecco's phosphate buffered saline (#806552‐500m; Sigma–Aldrich) and detached using 1× Trypsin/EDTA solution (# 59418C; Sigma–Aldrich). All murine cell lines were used in passages <30 for experiments.

The following human conventional cell lines were used: PaTu8988s (RRID:CVCL_1846), AsPC‐1 (RRID:CVCL_0152), MIA PaCa‐2 (RRID: CVCL_0428), HPAC (RRID: CVCL_3517), HuP‐T3 (RRID:CVCL_1299), Panc05.04 (RRID: CVCL_1637), HuP‐T4 (RRID:CVCL_1300). All human cell lines were cultivated in 1× RPMI 1640 GlutaMAX^‐I^ media (#61870010; Life Technologies) with 10% (v/v) FCS and 1% (v/v) penicillin/streptomycin. Human conventional PDAC cell lines were authenticated by STR profiling or multiplex human cell line authentication test (Multiplexion GmbH, Heidelberg, Germany). Mycoplasma contamination was tested by PCR‐based protocol as described recently [22]. The primary PDCLs were recently described [74] or isolated in the framework of the CRU5002 [75, 76]. These cells were cultured in RPMI with 20 % (v/v) FBS and 1 % (v/v) Penicillin/Streptomycin.

### 5.2 Patient‐Derived Organoids

Generation of PDOs from PDAC biopsies or tissues, propagation, and characterization was recently described [74]. PDO were cultured in the following medium: (DMEM‐F12 (#11320033; Thermo Fisher), 5mg/mL d‐glucose (#G8270; Sigma–Aldrich), 0.5% ITS Premix (#354350; Fisher Scientific), 5 nM 3,3,5‐triiodo‐l‐thyronine (#T0821; Sigma–Aldrich), 1 µM dexamethason (#D1756; Sigma Aldrich), 100 ng/mL Cholera Toxin (#C9903; Sigma–Aldrich), 1% penicillin–streptomycin (Thermo Fisher), 5% NU‐Serum IV (#355500; Fisher Scientific), 25 µg/mL bovine pituitary extract (#P1167; Sigma–Aldrich), 10 mM nicotinamide (#N3376; Sigma–Aldrich), 100 µg/mL Primocin (#ant‐pm05; Invivogen), 0.5 µm A83‐01 (#2939; Tocris), 10% RSPO1‐conditioned medium, 100 ng/mL recombinant human heregulin‐1 (#100‐03; Peprotech).

### 5.3 Statistics

All experiments were conducted in at least biological triplicates unless otherwise stated. In all figures, the mean and the standard deviation are depicted unless otherwise indicated. Analysis of variance (ANOVA), two‐sided Student's *t*‐test, Mann–Whitney *U*‐test, and Chi‐square test were used to analyze statistical significance, as indicated. *p* Values were corrected for multiple testing as indicated and determined with GraphPad Prism 5/8/7/9/10 (RRID:SCR_002798; GraphPad Software, California, USA). Tests were performed on non‐normalized data. *p* Values: unless indicated, *p < 0.05, **p < 0.01, ***p < 0.001, and ****p < 0.0001.

## Author Contributions

Conception and design of the study: JM, CS, ND, MW, OHK, PB, and RMS. Acquisition of data and/or analysis and interpretation of data: JM, CS, ND, HK, AR, XH, JB, CH, AN, DG, NR, LK, AN, SAW, CS, FO, RÖ, JJM, MD, ED, CTC, CJB, RMS, PB, MW, OHK, and GS. Generation of important models and contribution of essential resources, technology, and funding: JM, CS, HK, AR, XH, LK, BK, SM, SAW, RÖ, PK, VE, EH, MGr, MGh, CJB, RR, MR, UK, RMS, PB, DS, MW, OHK, and GS. Drafting of the manuscript: JM, CS, MW, OHK, and GS. Revision for important intellectual content: all authors. Approval of the final version for publication: all authors.

## Funding

Wilhelm‐Sander‐Stiftung grant 2019.086.1 (G.S. and O.H.K.) and grant 2023.027.1 (G.S., U.K., and M.W.); Deutsche Forschungsgemeinschaft (DFG) grant SCHN 959/6‐1 (G.S.), DFG grant SCHN959/7‐1 (G.S.), DFG grant SCHN959/8‐1 (G.S.), DFG grant SCHN959/11‐1 (G.S.), DFG grant KFO5002 (E.H., V.E, G.S.), DFG grant project 494535244 (U.K.), DFG grant project WI 6148/1‐1 (M.W.), Deutsche Krebshilfe DEFEAT‐PDAC consortium (M.R, R.R., D.S., G.S.), Deutsche Krebshilfe grant 70116474 (G.S.), Deutsche Krebshilfe grant 70115444 (M.W. and U.K.), Deutsche Krebshilfe grant 70114425 and 70114724 (U.K), Hector Stiftung grant M2408 (M.W.), and National Institutes of Health grant R01 GM141544 (P.L.B.).

## Ethics Statement

The primary human PDAC cellular models were established and analyzed in accordance with the declaration of Helsinki and approved by the local ethical committee TUM, Klinikum rechts der Isar (Project 207/15, 946/07, 330/19 and 80/17S) or University Medical Center Göttingen (UMG) (vote 11/5/17). The written informed consent from the patients for research use was obtained prior to the investigation.

## Conflicts of Interest

O.H.K. declares the patents WO2019/034538, WO2016020369A1, and WO/2004/027418, and paid advisory work for the BASF Ludwigshafen, Germany. WO2019/034538 (Synthesis, Pharmacology and use of New and Selective FMS‐like tyrosine kinase 3 (FLT3) FLT3 Inhibitors) covers substance classes that are discussed in this work. The substances that are covered in these patents are not the same that are shown in the submitted manuscript. The BASF has not influenced our study, and its products are not discussed in the manuscript. Thus, there are no direct competing interests. All other authors declare no conflicts of interest.

## Supporting information




**Figure S1**: Mesenchymal PDAC cells respond to LB100. (A) Inhibition of growth was measured by a clonogenic growth assay of murine mesenchymal (*n* = 4, 8248, 8513, 3250, S411) and epithelial (*n* = 3, 9591, 8296, S821) PDAC cell lines after treatment with 25 µM LB100 for 14 days (*p* = 0.057, two‐tailed Mann–Whitney test). Each dot represents one PDAC cell line and the mean of three independent biological replicates. Displayed is the percent inhibition of colony formation after treatment compared with the vehicle‐treated controls. (B) Representative bright field images of the mesenchymal and epithelial subpart of differential trypsinized Pank901m murine PDAC cell line after 5 and 10 µM of LB100 treatment (6 h). Scalebar = 100 µM. (C) Clonogenic growth assay in the mesenchymal and epithelial subpart from (B) after LB100 treatment with indicated concentrations for 14 days. One representative image of the experiment is displayed. (D) Quantification of four replicates from (C) (*n* = 4, two‐way ANOVA **p < 0.01, ****p < 0.0001). (E) LB100 dose–respond curves of separated mesenchymal (red) and epithelial part (blue) of a differential trypsinized F2612 murine PDAC cell line after treatment with LB100 in a 7‐point dilution or vehicle control for 3 days. Viability was measured by Cell‐Titer Glo assay. Dots represent the mean ± SD of at least three independent experiments. The GI_50_ values are indicated. (F) Representative picture of a clonogenic growth assay after treatment with indicated concentrations of LB100 from the cell line described in (E). (G) Quantification of four independent biological replicates from (F) (*n* = 4, *p value < 0.05, two‐tailed Mann–Whitney test). (H) Weekly representative bright field images from the in situ resistance assay over 5 weeks in the depicted PDCL lines with 0, 5, 10, or 20 µM LB100 treatment. Scalebar = 1 mm. (I) *Left*: Representative bright field images of the indicated primary PDCL from the CRU5002 cohort. *Right*: Cells were treated with LB100 in a 7‐point dilution for 72 h and assayed for cell viability (*n* ≥ 3). Displayed are the half‐maximal growth inhibitory (GI_50_) concentrations in epithelial and mesenchymal PDCL. (J) Population of mesenchymal murine PDAC cells with very high LB100 sensitivity was compared with murin mesenchymal PDAC cells with lower LB100 sensitivity by GSEA. GSEA was performed by GeneTrail3.2 using default settings but adding the HALLMARK signatures. Signatures were ranked by FDR and the top five five signatures were depicted. The enrichment score is color coded.
**Figure S2**: LB100‐induced cellular response. (A) Western blot analysis of phosphorylation of IRE1 and eIF2a in the indicated PDCL lines with increasing concentrations of LB100 for 6 h (0, 1, 5, 10, and 20 µM). HSP90: loading control (*n* = 1). (B) *Left*: Immunocytochemistry was used for visualizing stress granules via the TIA‐1 protein (red) after 4 h of treatment with LB100 (20 µM). Incubation with H_2_O_2_ (100 µM, 24 h) was used as a positive control (blue = DAPI staining). *Right*: Quantification of TIA‐1 stress granules per cell (TIA‐1/DAPI) from (b) from a minimum of three independent biological replicates of two mesenchymal (red, 8248 and 3250) and epithelial (blue, 9591 and 8296) cell lines. Each dot represents one quantified picture. (*p < 0.05, **p < 0.01, ***p < 0.001, ANOVA with Bonferroni correction). (C) *Left*: Western blot analysis of mesenchymal and epithelial cell lines after 6 h of treatment with LB100 (20 µM). Demonstrated is one representative western blot out of five independent experiments of the autophagy marker LC3 I/II. HSP90: loading control. *Right*: Quantification of LC3 II of the five independent experiments, normalized to mesenchymal control. *p < 0.05, two‐tailed paired *t*‐test. (D) Representative images of LysoTracker staining in the mesenchymal (8248) and epithelial (9591) PDAC cell line either treated with chloroquine (20 µM, 24 h) or LB100 (20 µM, 6 h) are shown. Displayed are the nuclei stainings via Hoechst 33342 (blue) and the lysosomes (LysoTracker Deep Red (L12492), green). Scalebar: 20 µm. (E) Quantification of the mean fluorescence intensity per cell from three independent biological experiments from (d). Each dot represents one quantified picture. (****p < 0.001, one‐way ANOVA with Bonferroni correction). (F) Depicted are the representative images from the LysoTracker staining as in a human LB100‐sensitive (red, MiaPaCa2) and resistant (blue, HUPT4) cell line. LB100 5 µM, 4 h, chloroquine 20 µM, 24 h. (G) Quantification of e and from additional staining's from the PDCL (PDC56 and PDC40, treatment as described in (f)) (one‐way ANOVA, ***p < 0.001, **p < 0.01, *n* = 2). (H) *Left*: Western blot analysis of p‐ULK1 (S757), p‐AMPK (T172), and pan AMPK after treatment with LB100 (20 µM, 6 h) or vehicle control. One representative blot out of minimum three independent experiments is shown. ß‐actin: loading control. *Right*: Quantification of the p‐ULK1 signal of five independent experiments (two‐way ANOVA, *p < 0.05).
**Figure S3**: CRISPR–Cas drop‐out screen. (A) and (B) Distribution of the calculated delta beta scores of the drop‐out screen in the murine PDAC cell line 9091 with (a) LB100 (10 µM) and (b) phendione (100 nM). Resistance: positive beta score, blue or synthetic lethal: negative beta score, red. (C) and (D) Delta beta scores of the screen were used as a rank for a preranked GSEA using the GeneTRAIL3 web interface and the GO‐BP signatures. (c) LB100 screen and (d) phendione screen. Blue: resistance, red: synthetic lethality.
**Figure S4**: Knockdown of *PPP2R1A* in MiaPaCa2–CasRX cells. (A) Western blot of PPP2R1A in parental MiaPaCa2, MiaPaCa2–CasRX, and MiaPaCa2–CasRX–gRNA–PPP2R1A cells transduced with a gRNA targeting PPP2R1A. Vinculin: loading control. (B) Quantification of (A). *n* = 3, **one‐way ANOVA *p* value < 0.01. (C) Relative growth of the cell lines described in (A) was determined over the indicated time points using cell titer glo assays. *Left*: 500 cells, *Middle*: 1000 cells, *Right*: 2000 cells were plated in three independent experiments. (D) *PPP2R1A* gene effects of a CRISPR–Cas9 drop‐out score (Chronos) for *n* = 48 PDAC cell lines were retrieved via the DepMap portal (DepMap Public 25Q3). (E) *PPP2R1A* mRNA expression log fold change of MiaPaCa2–CasRX and MiaPaCa2–CasRX–gRNA–PPP2R1A was computed in three independent replicates generated over 3 weeks. For each sample the log fold change is calculated and depicted. (F) and (G) Pathways connected to the LB100 Crispr drop‐out screen in MiaPaCa2 cells and genes deregulated upon *PPP2R1A* knockdown in MiaPaCa2–CasRX–gRNA–PPP2R1A cells. (F) GO‐BP‐terms, (G) HALLMARKS. (H) Overlapping pathways regulated by LB100 treatment and PPP2R1A knock‐down in MiaPaCa2 cells.
**Figure S5**: PP2A and the transcription cycle. (A) ssGSEA scores of the CPTAC PDAC data were retrieved via the *ProTrackPath: Pan‐cancer* web portal (normalization option: tumor and normal separately) and filtered for PDAC samples using the cBioPortal CPTAC clinical dataset. Variance‐scaled heatmap of the ssGSEA scores (clustering method = ward.D, clustering distance = euclidean) for the indicated HALLMARK signatures are depicted. (B) Kaplan–Meier survival curves from patients with high ssGSEA EMT signature scores (>75 percentile, red; events *n* = 23, censored *n* = 10) or low ssGSEA EMT scores (<75 percentile, blue; events *n* = 53, censored = 48). The *p* value of a log‐rank test is indicated. (C) Transcriptomes with high ssGSEA EMT signature scores (>75 percentile) or low ssGSEA EMT signature scores (<75 percentile) of the CPTAC PDAC RNAseq dataset were compared by a GSEA using Reactome signatures and the GeneTrail3.2 web portal. Significant (*q* < 0.05) signatures were filtered for the keywords “Polymerase II” and “transcription” and the results were illustrated. The enrichment score (ES) is color coded. (D) and (E) The PPP2CA protein expression of the CPTAC protein dataset (Prospective_CPTAC_PDAC, TMT MD abundance tumor) was accessed via the LinkedOmics web portal and queried for the phospho‐protein data (TMT MD abundance tumor); (D) analysis level: gene, (E) analysis level: site. (D) Overrepresentation analysis with the Pearson correlation coefficient and the FDR a rank selector for negatively correlating phospho‐proteins using GO BP analysis. (E) Pearson correlation coefficient for PPP2CA protein expression and phospho‐sites with a negative correlation coefficient (FDR<0.05) linked to transcription and transcriptional elongation. (F) RNA sequencing data of LB100‐treated MiaPaCa2 (6 h, 10 µM LB100) were compared with sequencing data after INTS8 knock‐out in HEK293 cells. Displayed are the significantly overlapping concordantly (blue) and discordantly (red) regulated genes. (G) RNA‐seq data of MiaPaCa2 cells treated for 6 h with 10 µM LB100 were analyzed by GSEA using the indicated signature and GeneTrail3.2. The enrichment blot is indicated and the *q*‐value depicted.
**Figure S6**: PP2a and regulation of small genes. (A) Length distribution of upregulated genes in murine PDAC cells (8248) with 2 h of LB100 treatment. (B) The correlation between gene length and the fold change in gene upregulation in murine PDAC cells (8248) after 2 h of LB100 treatment. (C) The enrichment of RNA polymerase II at different gene regions of 4 ER stress genes with varying lengths in murine PDAC cells (8248) with 2 h of LB100 treatment.

Supporting file1: mco270794‐sup‐0002‐TableS1.xlsx

Supporting file2: mco270794‐sup‐0003‐TableS2.xlsx

Supporting file3: mco270794‐sup‐0004‐TableS3.xlsx

## Data Availability

mRNA expression datasets of LB100‐treated mesenchymal (PPT‐8248, MiaPaCa2) and epithelial (PPT‐9591) cells can be freely accessed via the European Nucleotide Archive (ENA): PRJEB59091. mRNA expression datasets of gRNA‐PPP2R1A MiaPaCa2 cells can be accessed via Gene Expression Omnibus (GEO): GSE317590. ChIP‐seq data can be accessed via the GEO: GSE300008. All data needed to evaluate the conclusions in the paper are present in the paper and/or the Supporting Information.
