# Supplementary material for: The Protein Phosphatase Inhibitor LB100 Targets the Mesenchymal Lineage of Pancreatic Ductal Adenocarcinoma
Source: MedComm (2020). 2026 Jun 7;7(6):e70794. doi: 10.1002/mco2.70794 (PMC13243779; doi:10.1002/mco2.70794)
Supplement: Supplementary file 1 — Figure S1: Mesenchymal PDAC cells respond to LB100. (A) Inhibition of growth was measured by a clonogenic growth assay of murine mesenchymal (n = 4, 8248, 8513, 3250, S411) and epithelial (n = 3, 9591, 8296, S821) PDAC cell lines after treatment with 25 µM LB100 for 14 days (p = 0.057, two‐tailed Mann–Whitney test). Each dot represents one PDAC cell line and the mean of three independent biological replicates. Displayed is the percent inhibition of colony formation after treatment compared with the vehicle‐treated controls. (B) Representative bright field images of the mesenchymal and epithelial subpart of differential trypsinized Pank901m murine PDAC cell line after 5 and 10 µM of LB100 treatment (6 h). Scalebar = 100 µM. (C) Clonogenic growth assay in the mesenchymal and epithelial subpart from (B) after LB100 treatment with indicated concentrations for 14 days. One representative image of the experiment is displayed. (D) Quantification of four replicates from (C) (n = 4, two‐way ANOVA **p < 0.01, ****p < 0.0001). (E) LB100 dose–respond curves of separated mesenchymal (red) and epithelial part (blue) of a differential trypsinized F2612 murine PDAC cell line after treatment with LB100 in a 7‐point dilution or vehicle control for 3 days. Viability was measured by Cell‐Titer Glo assay. Dots represent the mean ± SD of at least three independent experiments. The GI50 values are indicated. (F) Representative picture of a clonogenic growth assay after treatment with indicated concentrations of LB100 from the cell line described in (E). (G) Quantification of four independent biological replicates from (F) (n = 4, *p value < 0.05, two‐tailed Mann–Whitney test). (H) Weekly representative bright field images from the in situ resistance assay over 5 weeks in the depicted PDCL lines with 0, 5, 10, or 20 µM LB100 treatment. Scalebar = 1 mm. (I) Left: Representative bright field images of the indicated primary PDCL from the CRU5002 cohort. Right: Cells were treated with LB100 in [file MCO2-7-e70794-s001.docx]

**Supplementary Information**

**The Protein Phosphatase Inhibitor LB100 Targets the Mesenchymal Lineage of Pancreatic Ductal Adenocarcinoma**

Janine Murr^1^, Carolin Schneider^2^, Ningjun Duan^2^, Hazal Köse^3^, Anantharamanan Rajamani^4^, Xueyang He^5,6,7^, Jonas Buchloh^2^, Christian Hintze^2^, Atharva Naik^2^, Daniel Goeke^1^, Nicole Rjasanow^2^, Lukas Krauß^2^, Alexandra Nguyen^8^, Sebastian A. Widholz^9^, Christian Schneeweis^4^, Riccardo Trozzo^9^, Felix Orben^1^, Sebastian Mueller^9^, Rupert Öllinger^9^, Juan J Montero^9^, Michael Dudek^10^, Percy Knolle^10^, Bo Kong^11^, Volker Ellenrieder^12,13,14^, Constanza Tapia Contreras^2^, Elisabeth Hessmann^12,13,14^, Marian Grade^2,14^, Michael Ghadimi^2,14^, Christian J. Braun^15^, Roland Rad^9,16,17^, Maximillian Reichert^1,16,17,18,19^, Ulrich Keller^3,16,20^, Roland M. Schmid^1^, Paul L. Boutz^5,6,7^, Dieter Saur^4,16,17^, Matthias Wirth^2,3,16,20^, Oliver H. Krämer^8$^, Günter Schneider^2,4,13,14,21*$^

^1^Medical Clinic and Polyclinic II, Klinikum rechts der Isar, Technical University Munich, Munich, 81675, Germany

^2^Department of General, Visceral and Pediatric Surgery, University Medical Center Göttingen, Göttingen, 37075, Germany

^3^Department of Hematology, Oncology and Cancer Immunology, Campus Benjamin Franklin, Charité-Universitätsmedizin Berlin, Corporate Member of Freie Universität Berlin and Humboldt-Universität zu Berlin, Berlin, Germany

^4^Institute for Translational Cancer Research and Experimental Cancer Therapy, Technical University Munich, 81675 Munich, Germany

^5^Department of Biochemistry and Biophysics, University of Rochester School of Medicine and Dentistry, Rochester, NY 14642, USA

^6^Center for RNA Biology, Rochester, NY 14642, USA

^7^Wilmot Cancer Institute, Rochester, NY 14642, USA

^8^Institute of Toxicology, University of Mainz Medical Center, Mainz, 55131, Germany

^9^Institute of Molecular Oncology and Functional Genomics, TUM School of Medicine, Technische Universität München, 81675 Munich, Germany

^10^Institute of Molecular Immunology and Experimental Oncology, University Hospital München rechts der Isar, Technical University of Munich, Ismaningerstr. 22, 81675 München Germany

^11^Department of General, Visceral and Transplantation Surgery, Heidelberg University Hospital, 6912 Heidelberg, Germany.

^12^University Medical Center Göttingen Department of Gastroenterology, Gastrointestinal Oncology and Endocrinology, 37075 Göttingen, Germany

^13^Clinical Research Unit 5002, KFO5002, University Medical Center Göttingen, 37075 Göttingen, Germany

^14^CCC-N (Comprehensive Cancer Center Lower Saxony), Göttingen, 37075, Germany

^15^Department of Pediatrics, Dr. von Hauner Children's Hospital, University Hospital, LMU Munich, Munich, 80337, Germany

^16^German Cancer Research Center (DKFZ) and German Cancer Consortium (DKTK), 69120 Heidelberg, Germany

^17^DEFEAT-PDAC – Decoding and targeting the PDAC ecosystem – a German Pancreatic Cancer Alliance (GPCA) consortium – partner site Munich

^18^Translational Pancreatic Research Cancer Center, Medical Clinic and Polyclinic II, Klinikum Rechts Der Isar, Technical University Munich, 81675 Munich, Germany

^19^Center for Organoid Systems (COS), TUM, Garching, Germany.

^20^Max Delbrück Center (MDC), 13125 Berlin, Germany

^21^DEFEAT-PDAC – Decoding and targeting the PDAC ecosystem – a German Pancreatic Cancer Alliance (GPCA) consortium – partner site Göttingen

*Running Title:* PP2A and pancreatic cancer

*^*^Corresponding author:* *Schneider, Günter*: University Medical Center Göttingen, Department of General, Visceral and Pediatric Surgery, 37075 Göttingen, Germany, phone: +49-5551-39-20488, e-mail: [guenter.schneider@med.uni-goettingen.de](mailto:guenter.schneider@med.uni-goettingen.de). *^$^equally contributing last authors.*

**Supplemental Figures**


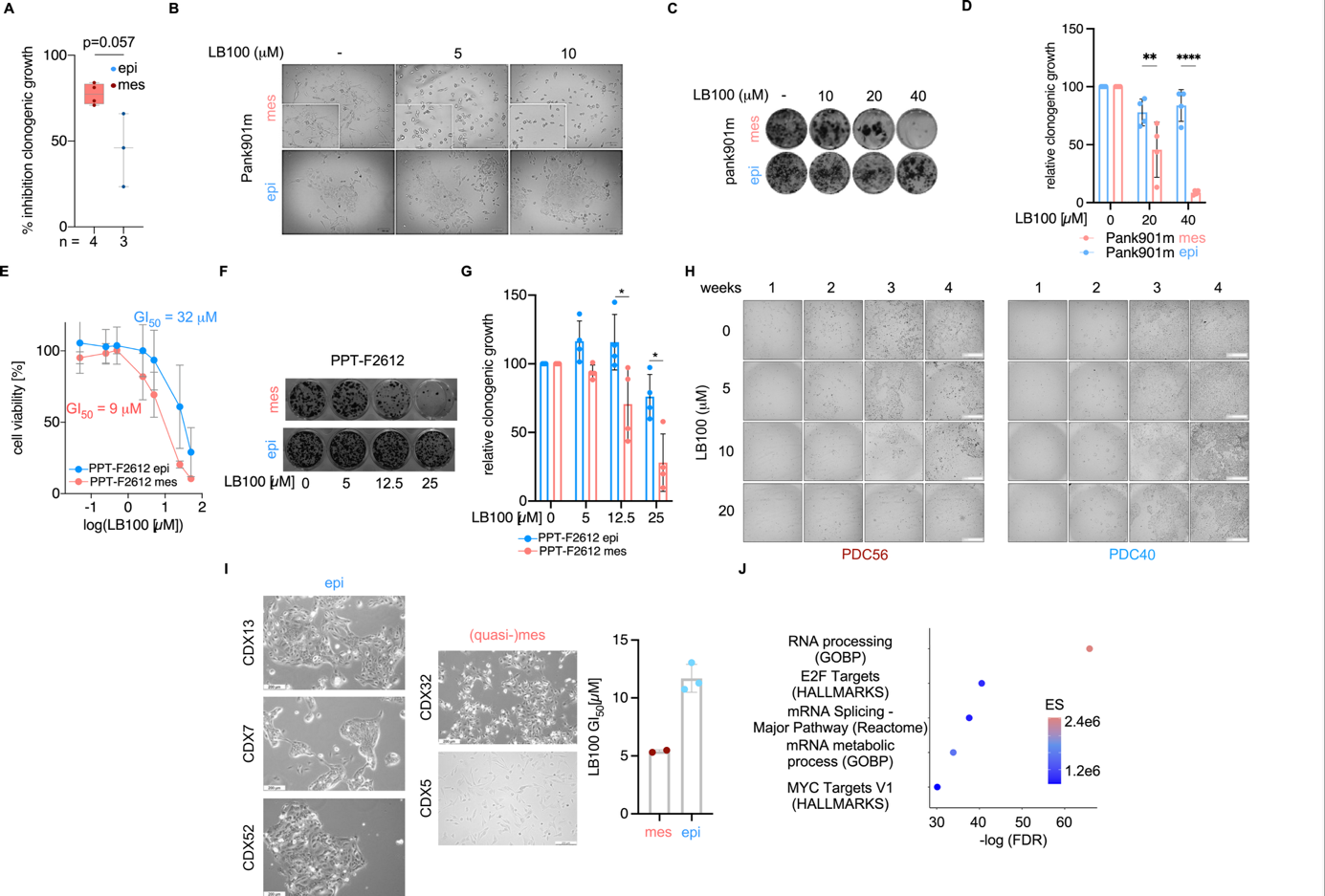


Figure S1 Mesenchymal PDAC cells respond to LB100

**A)** Inhibition of growth was measured by a clonogenic growth assay of murine mesenchymal (n = 4, 8248, 8513, 3250, S411) and epithelial (n = 3, 9591, 8296, S821) PDAC cell lines after treatment with 25 µM LB100 for 14 days (*p*= 0.057, two-tailed Mann Whitney test). Each dot represents one PDAC cell line and the mean of three independent biological replicates. Displayed is the percent inhibition of colony formation after treatment compared to the vehicle-treated controls. **B)** Representative bright field images of the mesenchymal and epithelial subpart of differential trypsinized Pank901m murine PDAC cell line after 5 and 10 µM of LB100 treatment (6 hours). Scalebar = 100 µM. **C)** Clonogenic growth assay in the mesenchymal and epithelial subpart from B) after LB100 treatment with indicated concentrations for 14 days. One representative image of the experiment is displayed. **D)** Quantification of four replicates from C) (n = 4, Two-Way ANOVA ***p*<0.01, ****p<0.0001). **E)** LB100 dose-respond curves of separated mesenchymal (red) and epithelial part (blue) of a differential trypsinized F2612 murine PDAC cell line after treatment with LB100 in a 7-point dilution or vehicle control for 3 days. Viability was measured by Cell-Titer Glo assay. Dots represent the mean +/- SD of at least three independent experiments. The GI_50_ values are indicated. **F)** Representative picture of a clonogenic growth assay after treatment with indicated concentrations of LB100 from the cell line described in E). **G)** Quantification of four independent biological replicates from F) (n = 4,*p value<0.05, two-tailed Mann-Whitney test). **H)** Weekly representative bright field images from the *in situ* resistance assay over 5 weeks in the depicted PDCL lines with 0, 5, 10 or 20 µM LB100 treatment. Scalebar = 1mm. **I)** *Left:* Representative bright field images of the indicated primary PDCL from the CRU5002 cohort. *Right:* cells were treated with LB100 in a 7-point dilution for 72h and assayed for cell viability (n ≥ 3). Displayed are the half-maximal growth inhibitory (GI_50_) concentrations in epithelial and mesenchymal PDCL. **J)** Population of mesenchymal murine PDAC cells with very high LB100 sensitivity was compared to murin mesenchymal PDAC cells with lower LB100 sensitivity by GSEA. GSEA was performed by GeneTrail3.2 using default settings but adding the HALLMARK signatures. Signatures were ranked by FDR and the top 5 five signatures were depicted. The enrichment score is color-coded.

**
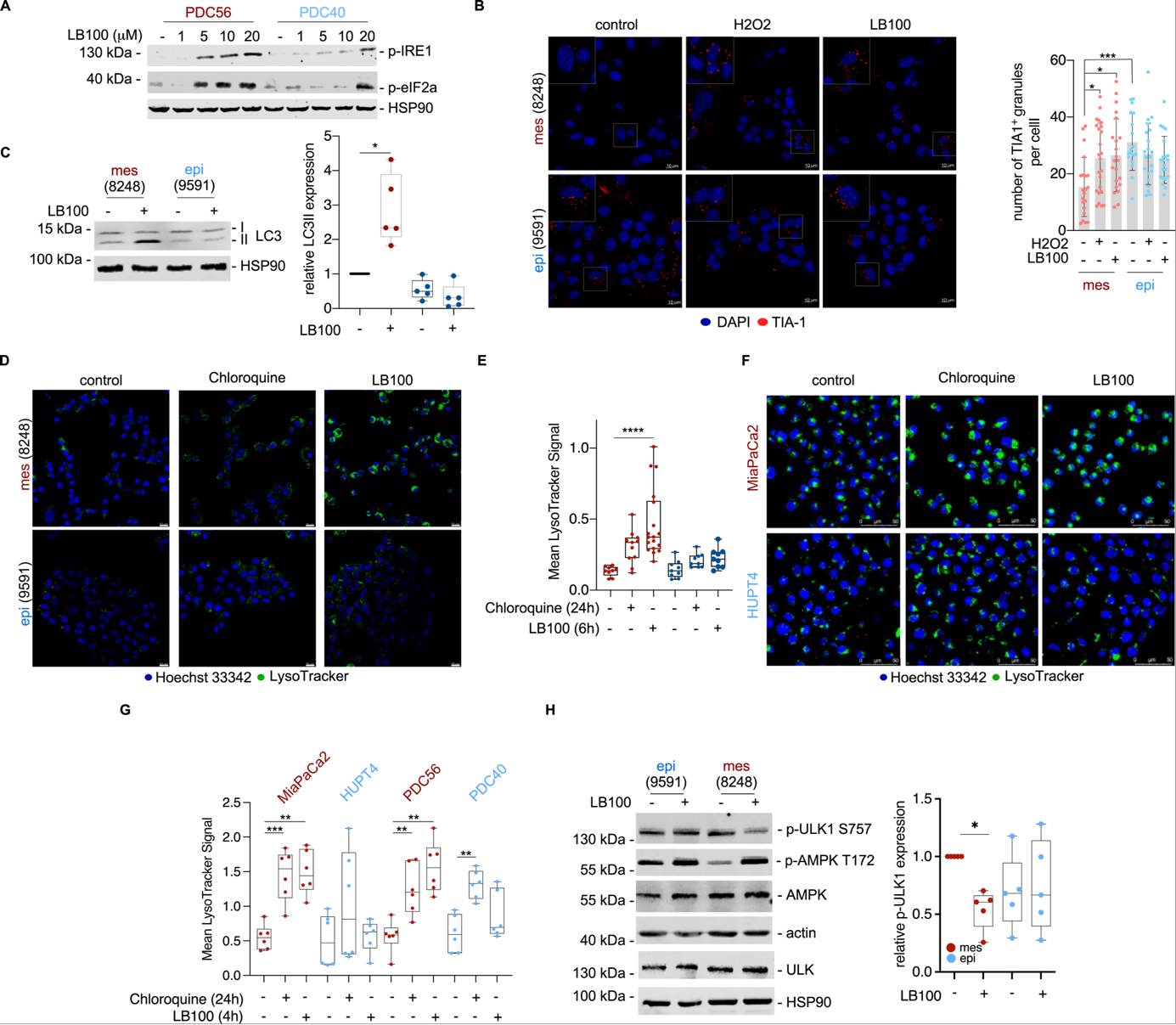
**

**Figure S2 LB100-induced cellular response**

**A)** Western Blot analysis of phosphorylation of IRE1 and eIF2a in the indicated PDCL-lines with increasing concentrations of LB100 for six hours (0 µ, 1 µM, 5 µM, 10 µM and 20 µM). HSP90: loading control (n = 1). **B)** *Left:* Immunocytochemistry was used for visualizing stress granules via the TIA-1 protein (red) after four hours of treatment with LB100 (20µM). Incubation with H2O2 (100µM, 24h) was used as a positive control (blue = DAPI staining). *Right:* Quantification of TIA-1 stress granules per cell (TIA-1/DAPI) from b) from a minimum of three independent biological replicates of two mesenchymal (red, 8248 and 3250) and epithelial (blue, 9591 and 8296) cell lines. Each dot represents one quantified picture. (* p<0.05, **p<0.01, ***p<0.001, ANOVA with Bonferroni correction). **C)** *Left:* Western Blot analysis of mesenchymal and epithelial cell lines after 6 hours of treatment with LB100 (20µM). Demonstrated is one representative western blot out of five independent experiments of the autophagy marker LC3 I/II. HSP90: loading control. *Right:* Quantification of LC3 II of the five independent experiments, normalized to mesenchymal control. *p<0.05, two-tailed paired t-test. **D)** Representative images of LysoTracker® staining in the mesenchymal (8248) and epithelial (9591) PDAC cell line either treated with Chloroquine (20 µM, 24 hours) or LB100 (20 µM, 6 hours) are shown. Displayed are the Nuclei stainings via Hoechst 33342 (blue) and the Lysosomes (LysoTracker Deep Red® (L12492), green). Scalebar: 20 µm. **E)** Quantification of the mean fluorescence intensity per cell from three independent biological experiments from d). Each dot represents one quantified picture. (****p<0.001, One-way ANOVA with Bonferroni correction). **F)** Depicted are the representative images from the LysoTracker staining as in a human LB100-sensitive (red, MiaPaCa2) and resistant (blue, HUPT4) cell line. LB100 5 µM, 4 hours, Chloroquine 20 µM, 24 hours. **G)** Quantification of e and from additional staining’s from the PDCL (PDC56 and PDC40, treatment as described in f)) (one-way ANOVA, ***p<0.001, **p<0.01, n = 2). **H)** *Left:* Western Blot Analysis of p-ULK1 (S757), p-AMPK (T172) and pan AMPK after treatment with LB100 (20µM, 6 hours) or vehicle control. One representative blot out of minimum three independent experiments is shown. ß-actin: loading control. *Right:* Quantification of the p-ULK1 signal of 5 independent experiments (Two-way ANOVA, * p<0.05).

**
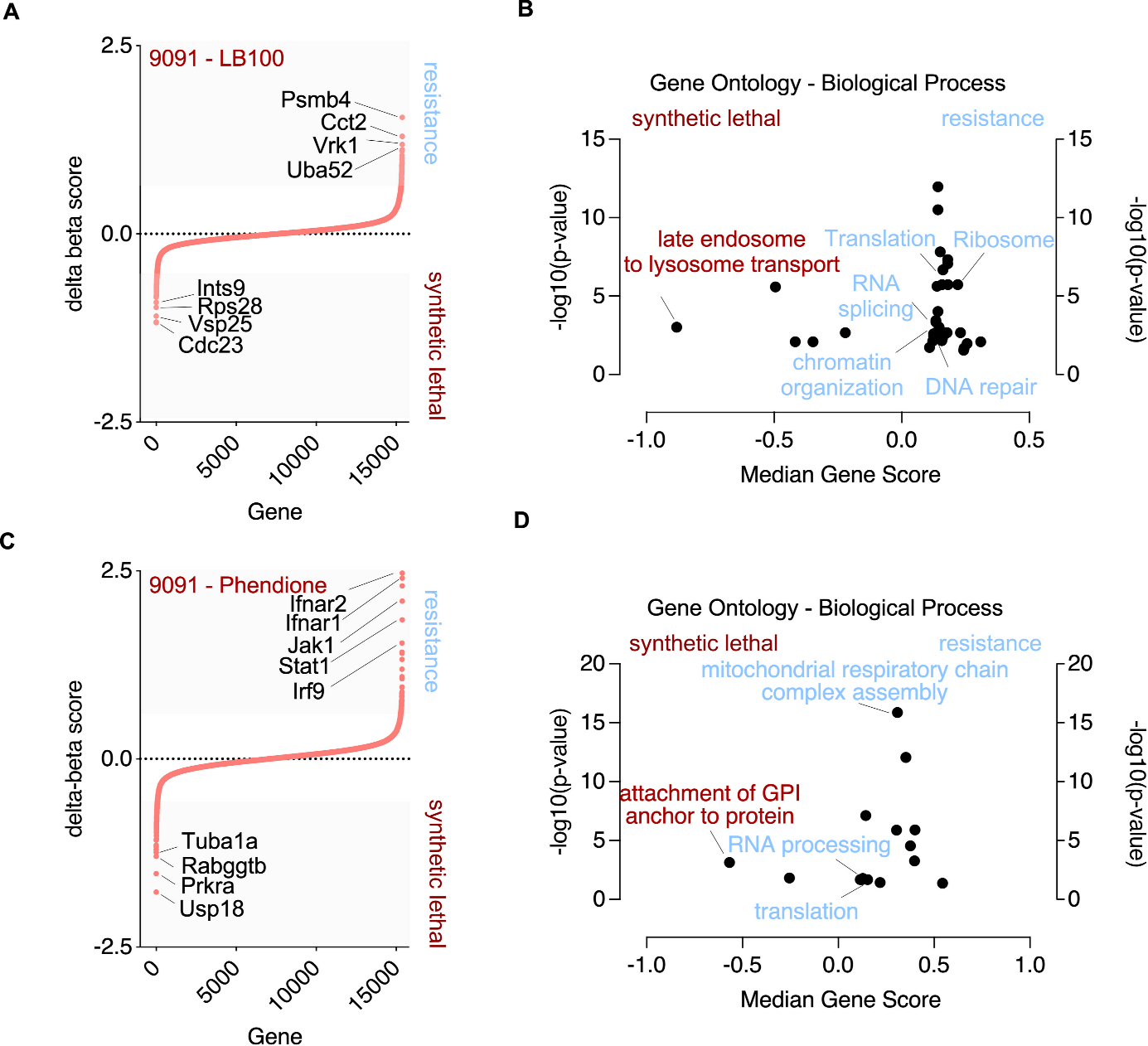
**

**Figure S3 CRISPR-Cas drop-out screen**

**A)** and **B)** Distribution of the calculated delta beta scores of the drop-out screen in the murine PDAC cell line 9091 with a) LB100 (10 µM) and b) Phendione (100 nM). Resistance: positive beta score, blue or synthetic lethal: negative beta score, red. **C)** and **D)** Delta beta scores of the screen were used as a rank for a pre-ranked GSEA using the GeneTRAIL3 web interface and the GO-BP signatures. c) LB100 screen, d) Phendione screen. Blue: resistance, Red: synthetic lethality.

**
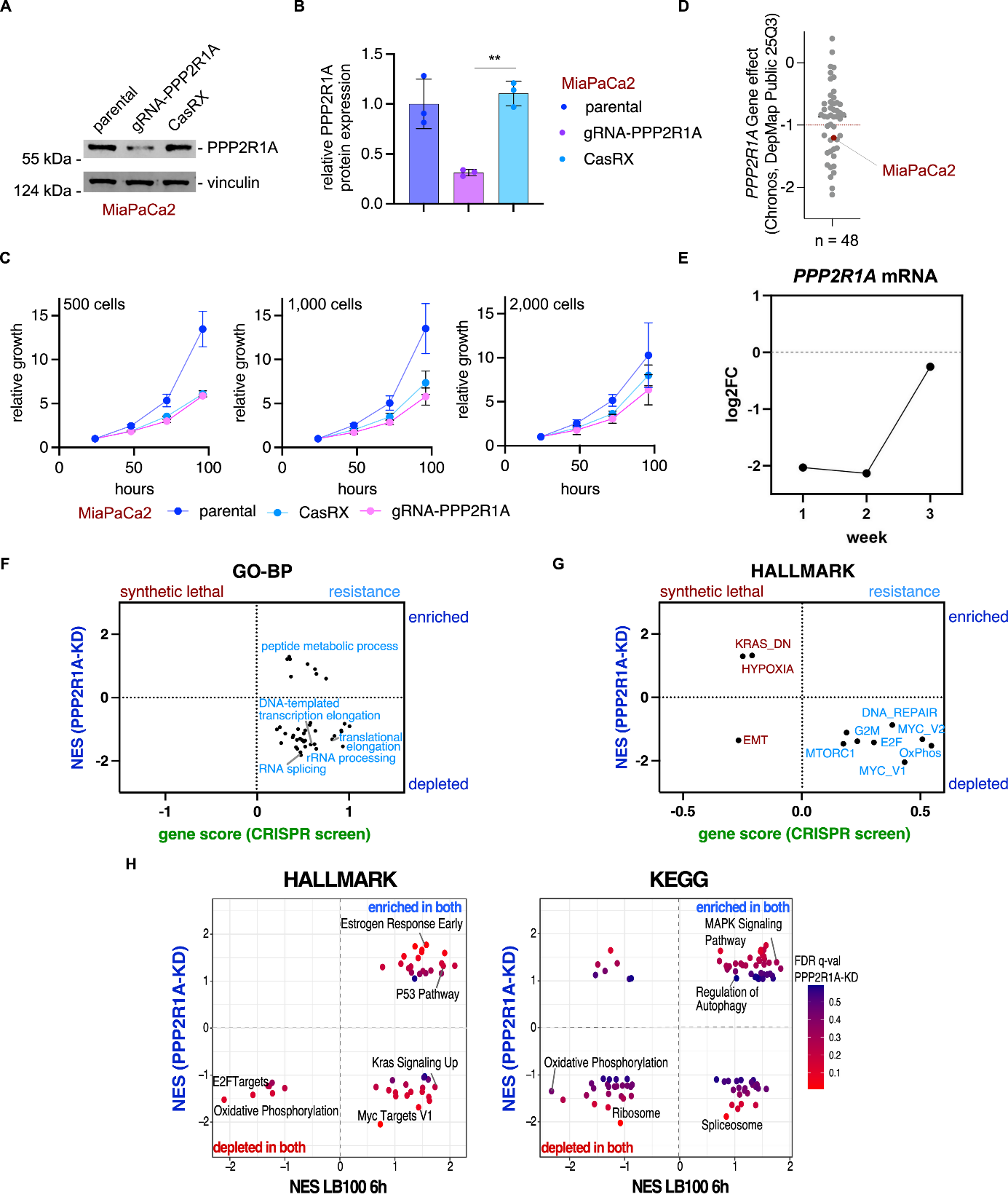
**

**Figure S4 Knockdown of *PPP2R1A* in MiaPaCa2-CasRX cells**

**A)** Western blot of PPP2R1A in parental MiaPaCa2, MiaPaCa2-CasRX, and MiaPaCa2-CasRX-gRNA-PPP2R1A cells transduced with a gRNA targeting PPP2R1A. Vinculin: loading control. **B)** Quantification of A). n = 3, **One-way ANOVA p value < 0.01. **C)** Relative growth of the cell lines described in A) was determined over the indicated time points using cell titer glo assays. Left: 500 cells, Middle: 1,000 cells, Right: 2,000 cells were plated in three independent experiments. **D)** *PPP2R1A* gene effects of a CRISPR-Cas9 drop-out score (Chronos) for n = 48 PDAC cell lines were retrieved via the DepMap portal (DepMap Public 25Q3). **E)** *PPP2R1A* mRNA expression log fold change of MiaPaCa2-CasRX and MiaPaCa2-CasRX-gRNA-PPP2R1A was computed in three independent replicates generated over three weeks. For each sample the log fold change is calculated and depicted. **F)** and **G)** pathways connected to the LB100 Crispr drop-out screen in MiaPaCa2 cells and genes deregulated upon *PPP2R1A* knockdown in MiaPaCa2-CasRX-gRNA-PPP2R1A cells. F) GO-BP-terms, G) HALLMARKS. **H)** Overlapping pathways regulated by LB100 treatment and PPP2R1A knock-down in MiaPaCa2 cells.

**
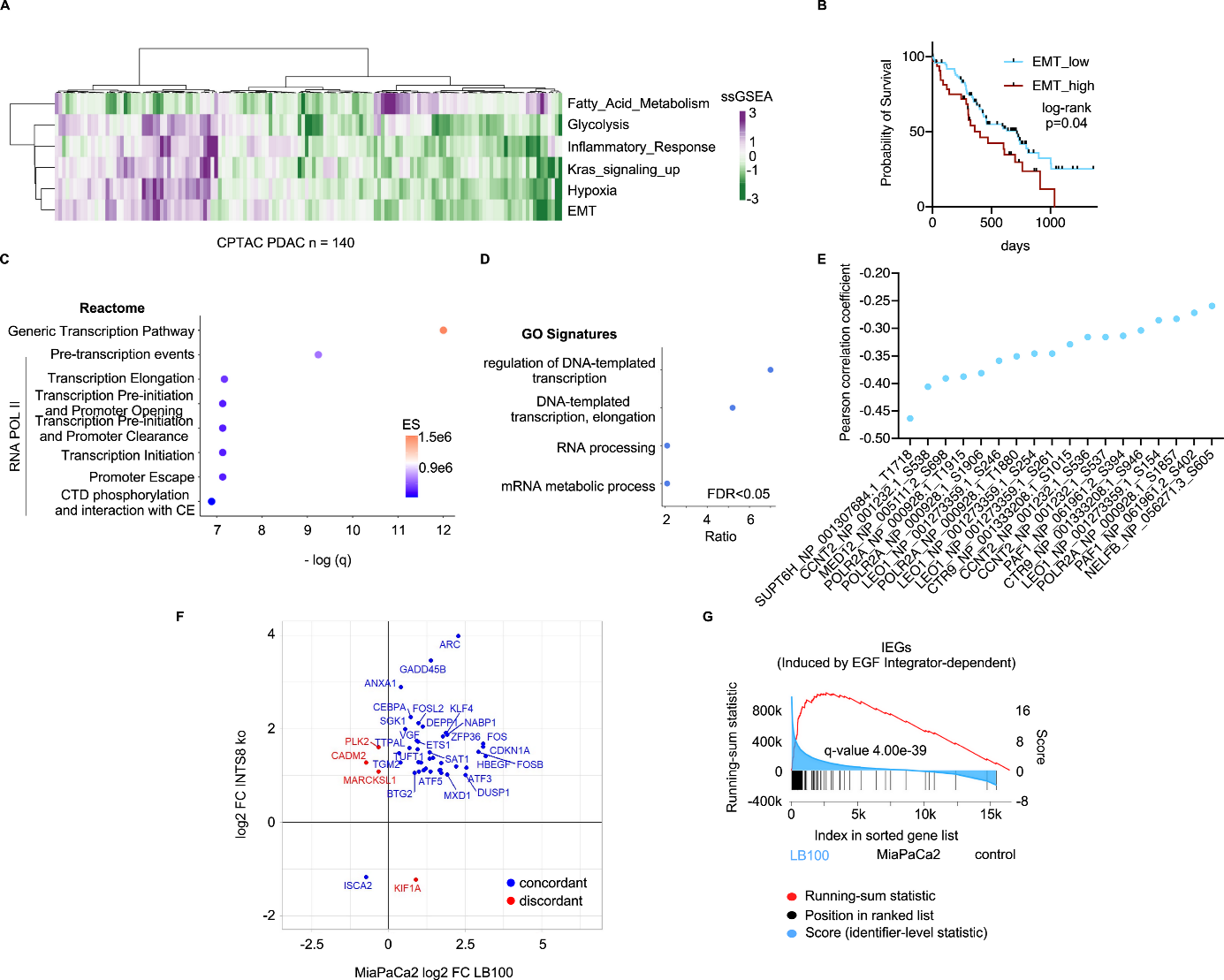
**

**Figure S5 PP2A and the transcription cycle**

**A)** ssGSEA scores of the CPTAC PDAC data were retrieved via the *ProTrackPath: Pan-cancer* web portal (normalization option: tumor and normal separately) and filtered for PDAC samples using the cBioPortal CPTAC clinical dataset. Variance-scaled heatmap of the ssGSEA scores (clustering method = ward.D, clustering distance = euclidean) for the indicated HALLMARK signatures are depicted. **B)** Kaplan Meier survival curves from patients with high ssGSEA EMT signature scores (>75 percentile, red; events n = 23, censored n = 10) or low ssGSEA EMT scores (<75 percentile, blue; events n = 53, censored = 48). The p-value of a log-rank test is indicated. **C)** Transcriptomes with high ssGSEA EMT signature scores (>75 percentile) or low ssGSEA EMT signature scores (<75 percentile) of the CPTAC PDAC RNAseq dataset were compared by a GSEA using Reactome signatures and the GeneTrail3.2 web portal. Significant (q<0.05) signatures were filtered for the keywords “Polymerase II” and “transcription” and the results were illustrated. The enrichment score (ES) is color-coded. **D)** and **E)** the PPP2CA protein expression of the CPTAC protein dataset (Prospective_CPTAC_PDAC, TMT MD abundance tumor) was accessed via the LinkedOmics web portal and queried for the phospho-protein data (TMT MD abundance tumor); D) analysis level: gene, E) analysis level: site. D) Overrepresentation analysis with the Pearson correlation coefficient and the FDR a rank selector for negatively correlating phospho-proteins using GO BP analysis. E) Pearson correlation coefficient for PPP2CA protein expression and phospho-sites with a negative correlation coefficient (FDR<0.05) linked to transcription and transcriptional elongation. **F)** RNA sequencing data of LB100-treated MiaPaCa2 (6 hours, 10 µM LB100) were compared with sequencing data after INTS8 knock-out in HEK293 cells. Displayed are the significantly overlapping concordantly (blue) and discordantly (red) regulated genes. **G)** RNA-seq data of MiaPaCa2 cells treated for 6 hours with 10 µM LB100 were analyzed by GSEA using the indicated signature and GeneTrail3.2. The enrichment blot is indicated and the q-value depicted.

**
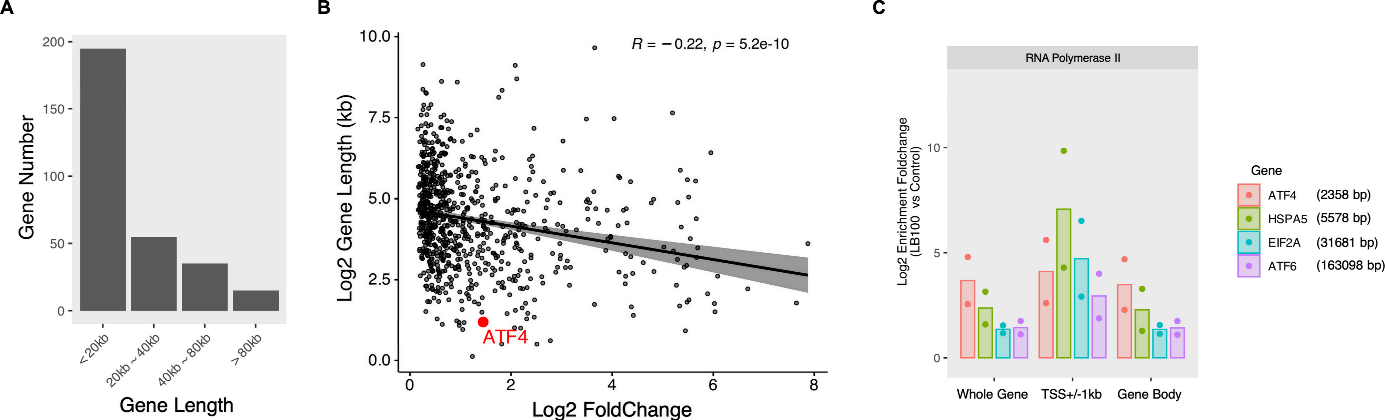
**

**Figure S6. PP2a and regulation of small genes**

**A)** Length distribution of upregulated genes in murine PDAC cells (8248) with 2 hours of LB100 treatment. **B)** The correlation between gene length and the fold change in gene upregulation in murine PDAC cells (8248) after 2 hours of LB100 treatment. **C)** The enrichment of RNA polymerase II at different gene regions of 4 ER stress genes with varying lengths in murine PDAC cells (8248) with 2 hours of LB100 treatment.

**Supplemental Material and Methods**

*Chemicals:*

LB100 (#S7537), Oligomycin A (#S1478), FCCP (#S8276), Cycloheximide (#S7418), Rotenon (#2348) ,SB-1317 (#S7002), and P276-00 (#S8058) were obtained from Selleckchem (Houston, TX, USA), 2-Deoxyglucose from Carl Roth (#CN96.3, Karlsruhe, Germany), Antimycin A (#A8674), 1,10-Phenanthrolin-5,6-dion (Phendione, #496383) and Chloroquine (#C6628) from Sigma-Aldrich (Taufkirchen, Germany).

*Generation of PPP2RA1 knock-down MiaPaCa2 cells by CasRX*

A stable *PPP2R1A* knockdown was generated using a previously described MiaPaCa2 cell line engineered with the Cas13d system, specifically the CasRx enzyme, delivered through a transposon-based system [1]. Single guide RNAs (sgRNAs) targeting *PPP2R1A* (Deposit 86760 in progress, ENST00000322088.6; crRNA2158: 2198–2220) were designed using Cas13designer [2,3]  (<https://cas13design.nygenome.org>). sgRNAs were cloned into the pLentiRNAGuide_001-hU6-RfxCas13d-DR1-BsmBI-EFS-Puro-WPRE vector containing a DsRed reporter (RRID:Addgene_138150). The folllowing oligonucleotides (a a a c G A A T A A A A T A A A A A C G G C A C A G T t t, a a a a a a A C T G T G C C G T T T T T A T T T T A T T C) were annealed using T4 polynucleotide kinase and ligated into the BsmBI-digested vector by Golden Gate cloning. Correct insertion was confirmed by agarose gel electrophoresis and Sanger sequencing. Lentiviral particles were produced in HEK293FT cells (RRID:CVCL_6911) by co-transfection of the sgRNA plasmid with psPAX2 (RRID:Addgene_12260) and pMD2.G (RRID:Addgene_12259) using TransIT-Lentiviral reagent. Viral supernatants were collected after four days, filtered (0.45 µm), and used to transduce MiaPaCa2 Cas13 [1]  cells by spin infection (1000 × g, 30 min, 33 °C) in the presence of polybrene (8 µg/ml). Puromycin selection (5 µg/ml) was initiated four days post-infection. Stable cell populations were identified by GFP/dsRed co-expression, and *PPP2R1A* knockdown efficiency was validated by Western blot analysis.

*Differential trypsinization*

Differential trypsinization of murine PDAC cell lines was done as described recently [4,5]. In brief, cell lines were differential trypsinized into their mesenchymal and epithelial fractions by different incubation times with 1x Trypsin/EDTA solution (# 59418C, Sigma-Aldrich). Cell media was aspirated and the cells washed one time with 1x PBS. A short incubation time of 2-3 minutes at room temperature with Trypsin/EDTA solution detached the mesenchymal subpart, whereas the epithelial fraction stayed attached. Both subparts were afterwards cultured separately and the procedure was then repeated for 3-6 times until stable, homogenous fractions (mesenchymal, epithelial) were enriched.

*CellTiter-Glo Viability assay*

For determining the cellular viability after inhibitor treatment in 2D cell lines, CellTiter-Glow® Luminescent Cell Viability Assay (#G7572, Promega, Walldorf, Germany) measurements were accomplished. For cell lines, 1,000-2,000 cells were seeded in 96 white, clear bottom plates (#3610, Corning Life Sciences, New York, USA), treated the next day with a 6- to 9-point dilution of LB100 (from 0.05-50 µM LB100) and incubated for 72 hours at 37°C, before adding 25 µl CellTiter-Glo reagent (Promega, #G7572) as endpoint measurement. Plate readings were done with a FluoStar Optima Luminescent Reader (BMG Labtech GmbH, Ortenberg, Germany). All experiments were done in biological and technical triplicates. The PDOs were screened for LB100 sensitivity and cell viability using a 384-well format and a 9-point LB100 dilution (from 0 - 200 uM LB100). Drug treatment in PDOs was recently described [6]. In brief, 500 cells were seeded in a 384-white well plate (#CLS3765, Corning Life Sciences) in drug screen media (Matrigel 1: 9 normal growth media + 10 µM Y-27632) and centrifuged (10 sec, 500rpm). Individual drugs were added in 4µl media/well the next day and after 72 hours, 5 µl CellTiter-Glo® Luminescent Cell Viability reagent (#G7572, Promega, Walldorf, Germany) was added to every single well. GI_50_ values were determined out of at least two independent biological replicates.

*GI_50_ calculation*

The half-maximal growth inhibitory concentration (GI_50,_ log(inhibitor) vs. normalized response) and the AUC were calculated with GraphPad Prism 9 (RRID: SCR_002798, GraphPad Software, California, V. 9.4.0), after normalizing to the control wells and log transformation.

*In-situ resistance assay.*

In-situ resistance assay was done as recently described [7]. In brief, 200 cells per well were seeded in 100 µl media in triplicates for each concentration (0, 5, 10 and 20 µM) on three plates for technical replicates (n=9). The next day, the indicated concentration of the drug was added in 100 µl media to the cells and confluency was checked after 72h with an Celigo imaging cytometer (Revvity, Hamburg, Germany). For the following 5 weeks, the media was changed to new drug containing media (200 µl) on the same day the week after as the initial treatment and always evaluated for confluency after 72h. Confluency at this time points was subjectively evaluated and a well was scored as resistant if the confluency was over 50%. After that, the probability of survival was determined for every well and a Kaplan-Meier survival curve was created. P values ware calculated using the log rank test.

*Clonogenic Assay*

2,000 cells were seeded into 24-well plates for Clonogenic Colony Formation assay, before treating the next day with the indicated concentrations of drugs. After 14 days, the media was aspirated, and the cells were washed one time with PBS and fixed with 0.2% (w/v) Crystal violet (Sigma-Aldrich, Darmstadt, Germany) solution in 2% (v/v) EtOH for 15 minutes. After washing three times with PBS, the fixed cells were dried overnight and imaged with a flatbed scanner. For quantification purposes, 600 µl of 1% (w/v) SDS-solution (Serva Electrophoresis GmbH, Heidelberg, Germany) was added and incubated, until no further clumps were visible. Absorbance at 595 nm was measured with a CLARIOstar microplate reader (BMG Labtech GmbH, Ortenberg, Germany). Values of a minimum of n = 3 independent biological experiments were calculated and normalized to each control.

*CRISPR resistance screen – human MiaPaCa2 cells*

Following the manufacturer’s protocol, 400ng of the library plasmid DNA [8] was amplified using ElectroMAX^TM^ D10HB cells (ThermoFisher, #18290015). Human Brunello CRISPR knockout pooled library was a gift from David Root and John Doench (Addgene #73178). The library was added and transferred into a chilled electroporation cuvette and pulsed (BioRad, Feldkirchen, Germany) at 1.8kV, (Ec1). SOC medium was directly added after the pulse and the cells were shaken in an incubator for 1h at 30°C. Cells were then spread on prewarmed LB-agar bioassay plates (ThermoFisher, #10489282) supplemented with Ampicillin 100ug/mL at 30°C for 16-18 hours. Transformants were pooled and plasmid DNA was extracted with the HiSpeed Plasmid Maxi Kit from Qiagen.

Lentivirus production was conducted by seeding HEK293T cells (4-5 million cells in 10cm dishes) in DMEM (ThermoFisher Gibco, #41965062) supplemented with 10% Fetal Bovine Serum (ThermoFisher Gibco, #A5256701). 1ml of OptiMEM (ThermoFisher Gibco, #31985-062) + 20µL of Lipofectamine2000 transfection reagent (ThermoFisher, #11668019) 10µg of each plasmid (pPAX2; pMD2G; lentiCas9-blast; sgRNA library). lentiCas9-Blast was a gift from Feng Zhang (Addgene plasmid # 52962) [9]. The mixture was incubated 20 minutes at room temperature and then added to the HEK293T cells and mixed gently. The medium was changed 12hours post-transfection and viral supernatant was collected 36 hours after transfection and filtered using a 0.45um syringe filter. A total of 3 million MiaPaCa2 cells were seeded in 6-well plates, one day prior to transduction. 3mL of the spCas9 viral supernatant were added to the cells with polybrene 1µg/mL (Merck, #TR-1003-G) and cells were spin-transduced for 1h at 2000 rpm at 32°C. 24 hours later, cells were split and selected with 10µg/mL Blasticidin S HCl (ThermoFisher Gibco, #A1113903). Cas9 expression was confirmed by western blot and functionality was assessed by sgRNA transfection. MiaPaCa2-Cas9 cells were seeded in 3 mL in 6-well plate at a density of 1million cells / ml. To determine the multiplicity of infection (MOI) of the virus, various dilutions were conducted in each well: 1:15, 1:30, 1:60, 1:120 and 1:240. 1µg/mL of polybrene was added (Merck, #TR-1003-G) and cells were centrifuged at 2000 rpm at 32°C for 1hour. One day after the spin-transduction, the cells were split in 2 groups and one group was selected with puromycin 1µg/µL (ThermiFisher, #A1113803). Calculation of the MOI: (Transfected cells treated with puromycin)/(Transfected cells without puromycin) x100)-(Untransfected cells treated with puromycin)/(Untransfected cells without puromycin) x100). Next, 3x10^8^ MiaPaCa2-Cas9 cells were seeded and transduced with the Brunello library which contains 19,112 targeted genes, 76,448 sgRNAs and 1000 controls aiming an MOI of 0.3. The transduction was conducted in the same conditions as the titer determination. The transduced cells were selected with 1µg/mL of puromycin for 7 days. Pellets were harvested and frozen for genomic DNA analysis on day 0. Cells were split into two groups, one treated with vehicle and the other with LB100 (5µM) for 14 days. At the end of the screen, cells were harvested for genomic DNA isolation (Macherey-Nagel, Tissue Kit, #740952.10). sgRNAs were amplified by PCR according to the manufacturer’s protocol, PCR products were purified using AMPpure beads and samples were sent to Novogene for next-generation sequencing (NGS).

*CRISPR resistance screen - murine 9091 cells*

For the library amplification, 100 ng of the murine BRIE CRISPR knockout pooled library [8] (a gift from David Root and John Doench (Addgene #73633, RRID: Addgene_73633) was added to a pre-chilled electroporation cuvette with 25 µl Endura ElectroCompetent cells (#71003-032, VWR International) and pulsed at 1.8 kV (EC1). Endura Rescue Media and additional SOC media was added to resuspending the cells and recovering at 250 rpm for 1h at 37°C, respectively. Bacteria were plated afterwards on LB-Amp Agar plates (100 µg/ml) overnight. To harvest the bacteria the next day, 10 ml of LB media were added on the plate and colonies were scrapped off with a cell spreader. To extract the plasmid DNA, bacteria were spinned down at 600 x g for 3 min and further processed with the NucleoBond Xtra Maxi Plus Plasmid Kit from Macherey Nagel (#740416.50, Macherey-Nagel). To produce the Lentivirus, 9.5 million HEK293FT (RRID: CVCL_6911) cells were plated in 15 cm plates in 20 ml DMEM media (10% FCS, 1% P/S). The next day, the transfection mix containing 1,900 µl Opti-MEM media (#31985062, Thermo Fisher Scientific), 15.6 µg psPAX2 plasmid (a gift from Didier Trono (Addgene plasmid # 12260 ; http://n2t.net/addgene:12260 ; RRID:Addgene_12260), 10.2 µg pMD2.G plasmid (a gift from Didier Trono (Addgene plasmid # 12259; http://n2t.net/addgene:12259 ; RRID:Addgene_12259) and 170 µl TransIT-LT1 transfection reagent (#MIR 2300, Mirus Bio LLC) were incubated for 30 min at room temperature before added dropwise to the cells. On day 3, the media was exchanged with 11 ml fresh media before collecting the viral supernatant on day 4. The viral supernatant was filtered with an 0.45 µM filter, aliquoted and further stored at -80°C. For the CRISPR resistance screen, a Cas9-expressing murine PDAC cell line (9091) (lentiCas9-Blast was a gift from Feng Zhang, Addgene plasmid # 52962; http://n2t.net/addgene:52962; RRID: Addgene_52962) was used. Cas9 expression and functionality was tested by western blot and sgRNA transfection before. 160 million cells per replicate and treatment condition were seeded in 6-well plates containing 3 million cells/well and spin-infected at 1000 x g for 2h at 33°C with a virus MOI of 0.3 and 500 x coverage of the 80.000 sgRNAs. 24h after the spin-infection, selection with 5 µg/ml Puromycin was started by splitting two wells in a 15 ml plate containing 20 ml media. After all cells died in the control (no virus added), the cells were recovered in normal growth media for additional 3 days. For the drug selection purpose, the respective GI_30_ concentrations were used (LB-100: 10 µM, Phendione: 100 nM). Drugs were replenished every 3-4 days for 14 days in total. After 14 days, the gDNA was extracted using the Blood & Cell culture DNA Maxi Kit (# 13362, Quiagen). To prep the library of the gDNA extraction with a 500x coverage, 228 µg gDNA per replicate was mixed with 76 µl forward primer (10 µM, P5), 76 µl reverse primer (10 µM, P7), 950 µl 2 x Kapa HIFI Short Master Mix (#08202923001, Roche) and water to 1.9 ml reaction volume. The volume was distributed to 38 reactions and amplified by PCR (95°C 3 min, (98°C 20 sec, 62°C 30 sec, 72°C 45 sec) x28, 72°C 5 min, 4°C). A 281 bp PCR product was visualized after agarose gel electrophoresis. 200 µl of the pooled product were cleaned with the Gel and PCR clean-up kit (#740609, Macherey-Nagel) with 5x more binding buffer and eluted in 25 µl H_2_O. Sequencing was performed exactly as described [10].

*Analysis of the Drop-out Screens*

Sequencing data were trimmed using the Trimmomatic tool [11] to maintain the base pairs containing the guide RNAs. The detection of enriched and depleted guides was performed using the MAGeCK mle algorithm 11 [12]. The sequenced library was used as a reference for the CRISPR screens in the murine 9091 line.

Subsequently, resulting delta beta scores were used as a rank to perform a pre-ranked GSEA analysis. A positive delta beta score reflected a positive selection of the gene under drug treatment, whereas a negative delta beta score means a gene is negatively selected, indicating synthetic lethality in combination with the drug. Here, Hallmark and Gene Ontology (Biological Process) pathways of the molecular signature database (MSigDb) were investigated using gene set enrichment analysis of GeneTrail [13].

*Deep Sequencing RNA-Seq analysis*

For the deep sequencing RNA-Sequencing, 300,000 cells were seeded in a 6-well format and treated the next day with the indicated concentrations of the inhibitors. After the incubation time (2 and 6 hours), the cells were washed one time with ice-cold PBS and homogenized in 1-Thioglycerol Homogenization solution from the Maxwell® RSC simplyRNA Tissue Kit (#AS1280, Promega, Walldorf, Germany). Library preparation and sequencing was performed by NGS (Novogene, United Kingdom). Briefly, mRNA was purified from total RNA using poly-T oligo-attached magnetic beads. After fragmentation, the first strand cDNA was synthesized using random hexamer primers followed by the second strand cDNA synthesis. The library was ready after end repair, A-tailing, adapter ligation, and size selection. After amplification and purification, insert size of the library was validated on an Agilent 2100 and quantified using quantitative PCR (Q-PCR). Libraries were then sequenced to a depth of 15 Gbp on Illumina NovaSeq 6000 S4 flowcell with PE150.

Raw RNA-seq reads were then mapped to the mouse mm10 genome build using STAR aligner version 2.5.3a (RRID:SCR_004463) [14] with the parameters: STAR --runMode alignReads --runThreadN 4 --genomeDir mm10 --twopassMode Basic --outSAMtype BAM SortedByCoordinate --outFilterMultimapNmax 20 --outFilterMismatchNmax 999 --outFilterMismatchNoverLmax 0.033 --alignIntronMin 70 --alignIntronMax 500000 --alignMatesGapMax 500000 --alignSJoverhangMin 8 --alignSJDBoverhangMin 1 --outSAMstrandField intronMotif --outFilterType BySJout. For additional details, see [15]. To identify detained introns, reads mapped with STAR aligner as described above were filtered for contiguous genome alignment. Mapped reads were then filtered using BedTools 2.25.0 (RRID:SCR_006646) [16] remove reads overlapping expressed repeats from the UCSC genome browser (RRID:SCR_005780)[29] RNA repeats and Ensembl mm10 annotations (RRID:SCR_002344) [17,18] and coding exons annotated in the Gencode mm10 (RRID:SCR_014966) annotations. Reads attributable to alternative polyadenylation sites were removed based on known Gencode polyadenylation sites. DESeq (RRID:SCR_000154) [19], was used to normalize intronic read counts and to account for coverage depth and dispersion across replicates. Intronic reads were partitioned and allocated to each intron proportional to its weight assuming all introns of a gene are present in equal abundance. Differential analysis using DESeq was then used to determine introns enriched in read coverage (observed read counts) compared to the expected counts using an FDR adjusted p-value threshold of 0.01 and fold change threshold of 2. We used custom Python (RRID:SCR_008394) scripts to derive a transcriptome annotation based on mm10 gene start and end boundaries, the genomic locations of detained introns as determined above, and the genomic locations of all splice junctions.

Detained introns: To quantify detained intron splicing differences between the conditions, 3 biological replicates were used for each treatment at each time point. The custom annotation described above was used as input to generate an ‘exon part’ gtf that is compatible with DEXSeq (RRID:SCR_012823) [20]. Differential expression of the alternative splicing events and detained introns was then determined using standard DEXSeq analysis, with a padj <0.05 as the cutoff for significant changes.

Alternative splicing: the published algorithm rMATs (RRID:SCR_001583) [21] was used to quantify skipped/cassette exons, alternative 3’ and 5’ splice sites, and mutually-exclusive exons. A significance padj of < 0.05 was used to determine significant splicing events.

Gene expression: The feature counts command from the subread software (RRID:SCR_009803) tool was used to produce the gene count matrix with the original bam files and input junction consensus gtf [22]. Differential gene expression was analyzed using DESeq2 (RRID:SCR_009803) between each treatment time and control samples. This derives a log2fold change and a corresponding adjusted p value for each gene in each treatment comparison. padj < 0.05 is used as cutoff for significant events.

*iRNAseq*

The above-described mRNA expression dataset was analyzed according to the published pipeline [23] using the Perl language.

*Overlap of LB100-treated and INTS8-depleted genexpression*

To find overlapping genes between the differential gene expression after two and six hours of LB100 treatment murine cell lines, the human MiaPaCa2 cell line and a previously published gene expression pattern after INTS8-depletion in Human HEK293T cells (Table S3) [24], the Gene ID were converted to mus musculus if necessary using the Orthology search tool (g:Orth) accessed from g:Profiler (<https://biit.cs.ut.ee/gprofiler/orth>). Afterwards, all significantly regulated genes (*p*< 0.05) in all datasets were compared, independently of their log2FC. Concordant genes can be found in both datasets up- or downregulated, discordant in only one of them.

*RNA polymerase II ChIP-seq*

ChIP-seq was performed using the SimpleChIP® Enzymatic Chromatin IP Kit (Magnetic Beads, Cell Signaling Technology, #9003) according to the manufacturer’s protocol. Briefly, 4 × 10⁶ cells were crosslinked with 1% formaldehyde for 10 min at room temperature and quenched with 0.125 M glycine for 5 min. Cells were then harvested and washed with cold PBS. After lysis, chromatin was digested with Micrococcal Nuclease for 20 min at 37 °C, followed by brief sonication. DNA was purified using spin columns.

Chromatin was diluted in ChIP buffer and incubated overnight at 4 °C with the total RNA Pol II CTD antibody (Abcam, ab26721). Immune complexes were captured using Protein G magnetic beads and washed, and DNA was eluted and purified using spin columns. The enriched DNA was stored at -20 °C until sequencing.

Raw paired-end ChIP-seq reads were first assessed for quality using FastQC (v0.12.1). High-quality reads were aligned to the mouse reference genome (GRCm39) using BWA-MEM (v0.7.18). Aligned reads were filtered and indexed with SAMtools (v1.21). Read enrichment at specific chromatin regions was quantified using featureCounts (v2.1.1). Coverage tracks were generated with bamCoverage (deepTools v3.5.6) and visualized in R using the Gviz package (v1.48.0). Differential binding analysis was conducted in R using DESeq2 (v1.44.0).

*Western Blot*

Western Blotting of whole cell lysates was done as described previously [25]. In Brief, cells were seeded in 10cm plates and treated the next day as indicated. After incubation, the supernatant was collected together with the trypsinized cells and lysed with RIPA buffer (1% (v/v) Triton-X, 1% (w/v) Na-deoxycholate, 0.1% (w/v) SDS, 150mM NaCl,10 mM EDTA and 20 mM TRIS-HCL, pH 7.5, supplemented with protease inhibitor and phosphatase inhibitor (#11873580001, Protease inhibitor cocktail complete EDTA free, Roche Diagnostics, Mannheim, Germany and Phosphatase-Inhibitor-Mix I, #39050, Serva Electrophoresis GmbH, Heidelberg, Germany) before freezing at -80°C overnight. After centrifugation at 4°C for 15 minutes (16.000 x g), the supernatant was used for a Bradford assay (#39222.03, Serva Electrophoresis GmbH, Heidelberg, Germany)) to determine the protein concentration. All samples were afterwards equality diluted in 5x Lämmli Loading buffer (45.6 mM Tris-HCl pH 6.8, 2% SDS, 10% glycerol, 1% β-mercaptoethanol, 0.01% bromophenol blue) before loading on a Polyacrylamide gel (7.5-15%) and separation for 3 hours at 80V. Blotting of the proteins to a 0.2µm Amersham™Protran™Nitrocellulose membrane (Merck-Millipore, Berlin, Germany) was done at 350mA for 1-2 hours with a wet blot system (Bio-Rad Laboratories Inc., Hercules, California, USA) before blocking the membrane in 5% (w/v) milkpowder (#T145.3, Carl Roth) in TBS-T. Primary antibodies were diluted 1:1000 in 5% (w/v) milk in TBST-T if not otherwise indicated and incubated overnight for 4°C. The following antibodies were used with the following dilutions: LC3 I/II (Cell Signaling Technology Cat# 4108, RRID:AB_2137703, 1:1000), LC3B (D11) XP (Cell Signaling Technology Cat#3868, RRID:AB_2137707, 1:1000), p-Ulk1 S757 (Cell Signaling Technology Cat# 6888, RRID:AB_10829226, 1:1000), p-AMPK T172 (Cell Signaling Technology Cat# 2535, RRID:AB_331250,1:1000), AMPK (Cell Signaling Technology Cat# 5832, RRID:AB_10624867,1:1000), p-eif2a (S51) (Cell Signaling Technology Cat# 3398, RRID:AB_2096481, 1:1000), ATF4 (Cell Signaling Technology Cat# 11815, RRID:AB_2616025, 1:1000), HSP90 (Santa Cruz Biotechnology Cat# sc-13119, RRID:AB_675659, 1:5000), p-S5 POL2R (Abcam Cat# ab5131, RRID:AB_449369, 1:2000), p-S2 POL2R (Abcam Cat# ab5095, RRID:AB_304749, 1:2000), pan-Pol II (Abcam Cat# ab264350, 1:2000), p-IRE1 (Abcam Cat# ab48187, RRID:AB_873899,1:1000), cleaved PARP (Cell Signaling Technology Cat# 5625, RRID:AB_10699459), ß-actin (Cell Signaling Technology Cat# 3700, RRID:AB_2242334, 1:5000), Vimentin (Cell Signaling Technology, Cat# 5741, RRID:AB_10695459, 1:1000), PP2A A Subunit (81G5) (Cell Signaling Technology Cat# 2041, RRID:AB_2168121, Dilution 1:1000 in 5% bovin serum albumin (BSA) (Sigma-Aldrich), Vinculin (Merck-Sigma-Aldrich Cat# V9131, RRID: AB_477629, Dilution 1:500). Secondary antibodies were used in a 1:10,000 dilution in 5% Milk-TBST and incubated for one hour at room temperature under gentle agitation: Mouse 680 (Cell Signaling Technology Cat# 5470, RRID:AB_10696895), Rabbit 680 (Cell Signaling Technology Cat# 5366, RRID:AB_10693812), Mouse 800 (Cell Signaling Technology Cat# 5257, RRID:AB_10693543) and Rabbit 800 (Cell Signaling Technology Cat# 5151, RRID:AB_10697505). Protein bands were visualized with an Odyssey® Imaging System (Licor Biosciences, Bad Homburg, Germany) at wavelength 700 or 800 nM, and the protein bands were quantified using the Image Studio Lite Software (RRID: SCR_013715, Licor Biosciences, Bad Homburg, Germany). All Protein quantifications were normalized to loading control of a minimum of three independent replicates.

*Flow cytometry Apoptosis detection via Annexin V/PI*

For the measurement of apoptosis, the FITC Annexin V Apoptosis Detection Kit I (RRID:AB_2869082, # 556547, BD Biosciences, San Diego, USA) was used. Cells were seeded in 6 ml culture media in 10 cm dishes and left for attachment for 24 hours. Cells were treated with 20 µM LB100 or left as untreated control. 6 hours after treatment the cells were washed with 4 ml PBS and detached with 1 ml of 0.05% (v/v) EDTA for 5 minutes. All media and cell washings steps were collected together with the detached cells and centrifugated for 5 minutes at 300 x g. The pellet was then resuspended in 500 µl 1x binding buffer with 5 µl Annexin and 2 µl PI, according to the manufacturer’s instructions. After 1 hour of staining, the samples were measured by a flow cytometer using CytoFLEX S (Beckman Coulter, California, USA). The obtained results from three biological replicates were afterwards analyzed using the FlowJo^TM^ Software (RRID: SCR_008520, FlowJo, LLC, Ashland, Oregon, USA).

*Autophagy Measurement by the LysoTracker®*

For measuring autophagy induction after inhibitor treatment, 10,000 cells were seeded into an 18-well µ-slide (Cat# 81816, Ibidi, Munich, Germany). The next day, treatment with the indicated concentrations of the inhibitors in human and murine cell lines was done for four or six hours, respectively. After the incubation time, the media was changed to LysoTracker® deep red media (50nM LysoTracker® in normal culture media) for one hour at 37°C, before adding 1 µg/µl Hoechst 33342 for 10 minutes (Invitrogen, California, USA). The cells were afterwards imaged via Live-cell imaging with a Leica SP8 Confocal microscope (Leica Biosystems,USA) under a constant 37°C temperature during the imaging process. Median Fluorescent intensity per cell was calculated with ImageJ (RRID:SCR_003070) and normalized to the Hoechst signal [26].

*Immunocytochemistry and imaging*

Cells were cultured on chamber slides and treated as indicated with the inhibitors. Afterwards, cells were fixed for 10 minutes with methanol-free 4% PFA (#28906 Thermo Scientific, USA), permeabilized with 0.1% (v/v) PBS-Triton X-100 and blocked with 1% (w/v) BSA / 10% (v/v) normal goat serum (#G9023, Sigma-Aldrich) / 0.3 M Glycin in 0.1 % (v/v) PBS-Tween for one hour. The primary antibody was incubated overnight at 4°C in 1 % (w/v) BSA solution in PBS. Used primary antibodies: TIA-1 (200 µg/ml, Santa Cruz Biotechnology Cat# sc-1751, RRID: AB_2201433). As a secondary antibody, Donkey anti-Goat IgG (H+L) Cross-Adsorbed Secondary Antibody, Alexa Fluor™ 555 (Thermo Fisher Scientific Cat# A-21432, RRID: AB_2535853) was used in a 1:1000 dilution in 1%(w/v) BSA solution in PBS for one hour. The cells were embedded in Vectashield HardSet Mounting media with DAPI (#H-1500 Vector Laboratories, Burlingame, USA) and imaged with a Leica SP8 Confocal microscope (Leica Camera AG, Wetzlar, Germany). TIA1+ granules on average per cell were calculated with Imaris v 8.3 (RRID: SCR_007370, Oxford Instruments, Abington, UK).

*Real-time cellular metabolism measurement*

One day prior to measurement, a Seahorse cartridge (#102416-100, Agilent Technologies, USA) was hydrated overnight, before changing to the calibration solution (#100840-000, Agilent Technologies, USA) 2h before the measurement. 10,000 cells were seeded in 80 µl Seahorse DMEM Media (#103334-100, Agilent Technologies, USA) and treated in quadruplicates for the indicated timepoints with the inhibitors. In Parallel, a 96 black well plate (#353219 Falcon, Corning Incorporated, NY, USA) was treated the same for later normalization by measuring Hoechst incorporation. After that, the media was changed to 180 µl normal Seahorse Media (pH 7.4) for ECAR measurements or to media containing 5 g/L Glucose for OCR measurements and incubated for 1 h at 37°C. The injection solutions for the OCR measurement were prepared in glucose-free Seahorse (pH 7.4) media as follows: Injection A (20 µl per injection) was used in a final well concentration of 2.5 µM Oligomycin, Injection B (22 µl per injection) contained 1 µM FCCP and 5 mM Pyruvate and Injection C (25 µl per injection) consisted of 2.5 µM Rotenone and 2.5 µM Antimycin A. The injection solutions for ECAR measurements were loaded as followed: Port A contained 10 mM Glucose, Port B 2.5 µM Oligomycin and Port C 100 mM 2-Deoxyglucose, all in a final well concentration respectively. Analyzation was done with a Seahorse XFe96 Analyzer (Agilent Technologies, USA). The parallel-prepared 96-well black plate was incubated with 1 µg/ml Hoechst 33342 solution for 5 minutes and then measured on a FLUOstar OPTIMA microplate reader (BMG Labtech GmbH, Ortenberg, Germany). Seahorse quadruplicates from each biological experiment (n≥3) were afterwards pooled, singularized and normalized to corresponding mean Hoechst 33342 values.

*Human and murine mRNA expression datasets, clinical data, PP2A dropout scores, Priority scores, ssGSEA of CPTAC, ssGSEA, LinkedOmics, GSEA, correlation of GI_50_ with gene expression, Venn analysis, and Heatmaps*

The target priority scores for the pancreatic cancer context were accessed via the project score I portal (<https://score.depmap.sanger.ac.uk/>) [27]. The portal has retired but source data can be accessed via <https://cellmodelpassports.sanger.ac.uk/> and scores are placed into Table S1. Priority score II data were accessed from [28]. For priority scores threshold of 40 was applied. CRISPR/Cas drop-out gene effects for PP2A in pancreatic cancer cell lines were accessed via the DepMap portal (<https://depmap.org>) [29]. mRNA expression dataset of 38 murine KRAS^G12D^-driven PDAC cell lines were described [4]. The murine expression dataset can be accessed via EBI European Nucleotide Archive: accession number PRJEB23787. The mRNA expression was quantified as log2 transcripts per million following correlation with the LB100 GI_50_ values using Pearson methods as recently described [30]. The Pearson correlation coefficient was used as a rank to provide a pre-ranked GSEA using the GSEA (GSEA app 4.2.3) using default settings and the signatures of the MolSigDB[31]. RNA sequencing of PDO was described [32] and ssGSEA was performed using Gene Pattern [33].

The Integrator-dependent EGF-induced IEGs were retrieved from [34] and used as a gene signature. The correlation of murine *Ppp2ca* mRNA with *Cdk9* mRNA was done in R-studio (2022.07.2) using the Pearson method and normalized mRNA data from [4]. The ssGSEA scores (normalization option: tumor and normal separately) of the PDAC CPTAC dataset [35,36] were directly accessed via the ProTrackPath: Pan-Cancer website (<http://pancan.cptac-data-view.org/>). Corresponding, RNA-seq data and clinical data were accessed and downloaded via the cBioPortal [37,38]. The analysis of the proteome and phospho-proteome data of the PDAC CPTAC [35] was performed via the LinkedOmics web portal (<https://www.linkedomics.org/login.php>) [39]. The Prospective_CPTAC_PDAC (n = 140) was used and the proteome data (platform: TMT MD abundance Tumor) was queried with the phospho-proteome data (TMT MD abundance Tumor, analysis level gene, or analysis level site). Overrepresentation analysis was performed with the GO BP signatures (Rank selection FDR, Direction: negatively correlated, significance level 0.05). Heatmaps, Venn Analysis, and illustration of data were done in GraphPad PRISM (5/8/7/9/10), R-studio (2022.07.2), ClustVis [40], and VENNY (bioinfogp.cnb.csic.es/tools/venny).

*References Supplemental Material & Methods*

1. Montero JJ, Trozzo R, Sugden M, et al. “Genome-scale pan-cancer interrogation of lncRNA dependencies using CasRx,” *Nat Methods* 21, no. 4 (2024):584-596.

2. Wessels HH, Méndez-Mancilla A, Guo X, Legut M, Daniloski Z, Sanjana NE. “Massively parallel Cas13 screens reveal principles for guide RNA design,” *Nat Biotechnol* 38, no. 6 (2020):722-727.

3. Guo X, Rahman JA, Wessels HH, et al. “Transcriptome-wide Cas13 guide RNA design for model organisms and viral RNA pathogens,” *Cell Genom* 1, no. 1 (2021):100001.

4. Mueller S, Engleitner T, Maresch R, et al. “Evolutionary routes and KRAS dosage define pancreatic cancer phenotypes,” *Nature* 554, no. 7690 (2018):62-68.

5. Krauß L, Urban BC, Hastreiter S, et al. “HDAC2 facilitates pancreatic cancer metastasis,” *Cancer Res* 82, no. 4. (2022): 695-707.

6. Orben F, Lankes K, Schneeweis C, et al. “Epigenetic drug screening defines a PRMT5 inhibitor sensitive pancreatic cancer subtype,” *Jci Insight* 7, no. 10 (2022): e151353.

7. Sealover NE, Theard PT, Hughes JM, Linke AJ, Daley BR, Kortum RL. “In situ modeling of acquired resistance to RTK/RAS-pathway-targeted therapies,” *iScience* 27, no. 1 (2024):108711.

8. Doench JG, Fusi N, Sullender M, et al. “Optimized sgRNA design to maximize activity and minimize off-target effects of CRISPR-Cas9,” *Nat Biotechnol* 34, no. 2 (2016):184-191.

9. Sanjana NE, Shalem O, Zhang F. “Improved vectors and genome-wide libraries for CRISPR screening,” *Nat Methods* 11, no. 8 (2014):783-784.

10. Griger J, Widholz SA, Jesinghaus M, et al. “An integrated cellular and molecular model of gastric neuroendocrine cancer evolution highlights therapeutic targets,” *Cancer Cell* 41, no. 7 (2023):1327-1344.e10.

11. Bolger AM, Lohse M, Usadel B. “Trimmomatic: a flexible trimmer for Illumina sequence data,” *Bioinformatics* 30, no. 15 (2014):2114-2120.

12. Li W, Xu H, Xiao T, et al. “MAGeCK enables robust identification of essential genes from genome-scale CRISPR/Cas9 knockout screens,” *Genome Biol* 15, no. 12 (2014):554.

13. Gerstner N, Kehl T, Lenhof K, et al. “GeneTrail 3: advanced high-throughput enrichment analysis,” *Nucleic Acids Res* 48, W1 (2020):gkaa306-.

14. Dobin A, Davis CA, Schlesinger F, et al. “STAR: ultrafast universal RNA-seq aligner,” *Bioinformatics* 29, no. 1 (2013):15-21.

15. Boutz PL, Bhutkar A, Sharp PA. “Detained introns are a novel, widespread class of post-transcriptionally spliced introns,” *Gene Dev* 29, no. 1 (2015):63-80.

16. Quinlan AR, Hall IM. “BEDTools: a flexible suite of utilities for comparing genomic features,” *Bioinformatics* 26, no. 6 (2010): 841-842.

17. Yates A, Akanni W, Amode MR, et al. “Ensembl 2016,” *Nucleic Acids Res* 44, no. D1 (2016):D710-D716.

18. Harrow J, Frankish A, Gonzalez JM, et al. “GENCODE: The reference human genome annotation for The ENCODE Project,” *Genome Res* 22, no. 9 (2012):1760-1774.

19. Anders S, Huber W. “Differential expression analysis for sequence count data,” *Genome Biol* 11, no. 10 (2010):R106.

20. Anders S, Reyes A, Huber W. “Detecting differential usage of exons from RNA-seq data,” *Genome Res* 22, no. 10 (2012):2008-2017.

21. Park JW, Tokheim C, Shen S, Xing Y. “Deep Sequencing Data Analysis,” *Methods Mol Biology* 1038, (2013):171-179.

22. Liao Y, Smyth GK, Shi W. “The Subread aligner: fast, accurate and scalable read mapping by seed-and-vote,” *Nucleic Acids Res* 41, no.10 (2013):e108-e108.

23. Madsen JGS, Schmidt SF, Larsen BD, Loft A, Nielsen R, Mandrup S. “iRNA-seq: computational method for genome-wide assessment of acute transcriptional regulation from total RNA-seq data,” *Nucleic Acids Res* 43, no. 6 (2015): e40-e40.

24. Huang KL, Jee D, Stein CB, et al. “Integrator Recruits Protein Phosphatase 2A to Prevent Pause Release and Facilitate Transcription Termination,” *Mol Cell* 80, no. 2 (2020): 345-358.e9.

25. Schneeweis C, Diersch S, Hassan Z, et al. “AP1/Fra1 confers resistance to MAPK cascade inhibition in pancreatic cancer,” *Cell Mol Life Sci* 80, no. 1 (2023):12.

26. Schneider CA, Rasband WS, Eliceiri KW. “NIH Image to ImageJ: 25 years of image analysis,” *Nat Methods* 9, no. 7 (2012):671-675.

27. Behan FM, Iorio F, Picco G, et al. “Prioritization of cancer therapeutic targets using CRISPR–Cas9 screens,” *Nature*. 568, no. 7753 (2019): 511-516.

28. Pacini C, Duncan E, Gonçalves E, et al. “A comprehensive clinically informed map of dependencies in cancer cells and framework for target prioritization,” *Cancer Cell* 43, no. 2 (2024):301-316.

29. Dempster JM, Boyle I, Vazquez F, et al. “Chronos: a cell population dynamics model of CRISPR experiments that improves inference of gene fitness effects,” *Genome Biol* 22, no. 1 (2021): 343.

30. Lier S, Sellmer A, Orben F, et al. “A novel Cereblon E3 ligase modulator with antitumor activity in gastrointestinal cancer,” *Bioorg Chem* 119, (2022): 105505.

31. Subramanian A, Tamayo P, Mootha VK, et al. “Gene set enrichment analysis: a knowledge-based approach for interpreting genome-wide expression profiles,” *P Natl Acad Sci Usa*. 102, no. 43 (2005):15545-15550.

32. Peschke K, Jakubowsky H, Schäfer A, et al. “Identification of treatment‐induced vulnerabilities in pancreatic cancer patients using functional model systems,” *Embo Mol Med* 14, no. 4 (2022): e14876.

33. Reich M, Liefeld T, Gould J, Lerner J, Tamayo P, Mesirov JP. “GenePattern 2.0.,” *Nat Genetics* 38, no. 5 (2006):500-501.

34. Yue J, Lai F, Beckedorff F, Zhang A, Pastori C, Shiekhattar R. “Integrator orchestrates RAS/ERK1/2 signaling transcriptional programs,” *Gene Dev* 31, no. 17 (2017):1809-1820.

35. Cao L, Huang C, Zhou DC, et al. “Proteogenomic characterization of pancreatic ductal adenocarcinoma,” *Cell* 184, no. 19 (2021):5031-5052.e26

36. Liang WW, Lu RJH, Jayasinghe RG, et al. “Integrative multi-omic cancer profiling reveals DNA methylation patterns associated with therapeutic vulnerability and cell-of-origin,” *Cancer Cell* 41, no. 9, (2023): 1567-1585.

37. Gao J, Aksoy BA, Dogrusoz U, et al. “Integrative Analysis of Complex Cancer Genomics and Clinical Profiles Using the cBioPortal,” *Sci Signal* 6, no. 269 (2013):pl1.

38. Cerami E, Gao J, Dogrusoz U, et al. “The cBio Cancer Genomics Portal: An Open Platform for Exploring Multidimensional Cancer Genomics Data,” *Cancer Discov* 2, no. 5 (2012):401-404.

39. Vasaikar SV, Straub P, Wang J, Zhang B. “LinkedOmics: analyzing multi-omics data within and across 32 cancer types,” *Nucleic Acids Res* 46, (Database issue) (2017):gkx1090-.

40. Metsalu T, Vilo J. “ClustVis: a web tool for visualizing clustering of multivariate data using Principal Component Analysis and heatmap,” *Nucleic Acids Res* 43, no. W1 (2015): W566-W570. doi:10.1093/nar/gkv468
